# Advances in haemostatic sponges: Characteristics and the underlying mechanisms for rapid haemostasis

**DOI:** 10.1016/j.bioactmat.2023.04.008

**Published:** 2023-04-13

**Authors:** Akriti Nepal, Huong D.N. Tran, Nam-Trung Nguyen, Hang Thu Ta

**Affiliations:** a Queensland Micro-and Nanotechnology Centre, Griffith University, Nathan, Queensland, 4111, Australia; b Australian Institute for Bioengineering and Nanotechnology, University of Queensland, St Lucia, Queensland, 4072, Australia; c Bioscience Discipline, School of Environment and Science, Griffith University, Nathan, Queensland, 4111, Australia

**Keywords:** Haemostatic sponge, Characteristics, Mechanism, Haemostasis, Nanoparticles

## Abstract

In traumatized patients, the primary cause of mortality is uncontrollable continuous bleeding and unexpected intraoperative bleeding which is likely to increase the risk of complications and surgical failure. High expansion sponges are effective clinical practice for the treatment of wound bleeding (irregular/deep/narrow) that are caused by capillaries, veins and even arterioles as they possess a high liquid absorption ratio so can absorb blood platelets easily in comparison with traditional haemostasis treatments, which involve compression, ligation, or electrical coagulation etc. When in contact with blood, haemostatic sponges can cause platelet adhesion, aggregation, and thrombosis, preventing blood from flowing out from wounds, triggering the release of coagulation factors, causing the blood to form a stable polymerized fibre protein, forming blood clots, and achieving the goal of wound bleeding control. Haemostatic sponges are found in a variety of shapes and sizes. The aim of this review is to facilitate an overview of recent research around haemostatic sponge materials, products, and technology. This paper reviews the synthesis, properties, and characteristics of haemostatic sponges, together with the haemostasis mechanisms of haemostatic sponges (composite materials), such as chitosan, cellulose, gelatin, starch, graphene oxide, hyaluronic acid, alginate, polyethylene glycol, silk fibroin, synthetic polymers silver nanoparticles, zinc oxide nanoparticles, mesoporous silica nanoparticles, and silica nanoparticles. Also, this paper reviews commercial sponges and their properties. In addition to this, we discuss various in-vitro/in-vivo approaches for the evaluation of the effect of sponges on haemostasis.

## Highlights

• High expansion sponges quickly absorb blood platelets, stopping bleeding from irregular, deep, and narrow wounds.
• Natural polymers have been used as haemostatic sponge substrates.
• Synthetic polymers are gaining favour because they can mitigate the problems associated with natural polymers.
• Nanoparticles are incorporated into sponges to enhance mechanical strength, haemostatic and antibacterial capabilities.
• Testing methods and animal models vary amongst studies, thus challenging to compare effectiveness of the developed sponges.

## Graphical abstract

Mostly used materials and nanoparticles to develop haemostatic sponges.

**Figure:**
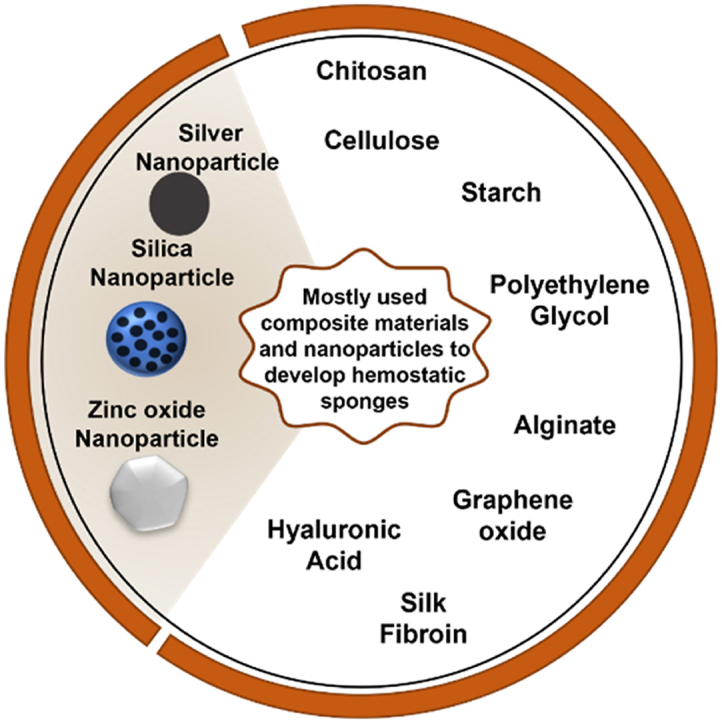

## 1 Introduction

The leading cause of death in traumatized patients is due to uncontrollable continuous bleeding, and an increase in complication rates and the likelihood of surgical failure is due to unexpected intraoperative bleeding [1]. Major pathological implications and sequelae that directly increase physical morbidity and death are caused by massive bleeding, which also includes metabolic and cellular dysfunction [2,3], haemorrhagic shock, coagulation, and acidosis [4]. Since 2000, several types of research have been conducted to advance bleeding-control technology and to investigate new haemostatic and absorptive agents with increasing proof of their efficiency as compared to gauze dressings [5]. Hydrogels, sponges, microspheres, membranes and tourniquet bandages are different haemostatic materials used widely in recent developments in the medical industry [[6], [7], [8]]. Haemostatic agents should ideally include characteristics like biocompatibility and stability, ability to promote the healing process of tissues without negative effects, be readily useable, lightweight, non-toxic/irritant and affordability, including the ability to control major haemorrhage from large arteries, veins, and visceral organs quickly. In past years, several haemostatic sponges have been developed aimed at increasing the survival rate and reducing bleeding problems in wounded patients [9].

A haemostatic sponge has absorbent and antiseptic properties and stimulates the tissue regeneration [10]. As a tool, the sponge is used in surgery, neurosurgery, dentistry, otolaryngology, and gynaecology to stop blood loss and to close wound surfaces (burns, trophic ulcers) [11,12]. Haemostatic sponges help to absorb a large amount of liquid from the blood as they are porous in their structure, and able to concentrate coagulation factors, red blood cells, and platelets for a more efficient clotting process [1]. Today, cellulose and hemicellulose, gelatin, alginate, chitosan, starch, and other polymeric materials can be utilised to make medical/haemostatic sponges [[13], [14], [15]]. The first medical sponges were made from collagen, gelatin, and oxycellulose, with no extra coatings or active pharmacological components added [16]. To boost their haemostatic efficiency and seal the tissues, the sponges were coated with fibrinogen and thrombin to provide sticky surfaces for the wound. Synthetic or protein-reactive sealant components are also utilised. Collagen coated with polyethylene glycol (PEG), or oxidized cellulose coated with PEG are two new haemostatic sponges that have recently been created and utilised in surgical practice [17]. According to research conducted by the European Association of Cardiothoracic Surgery, “CoSeal®" is a successful technique for avoiding pericardial adhesions in patients who may require surgery, particularly infants with congenital heart disease. “CoSeal®" is made up of two biocompatible PEG mixed with a weak hydrogen chloride solution to generate a covalently bonded hydrogel that adheres to both tissue and synthetic grafts [18]. In addition, a considerable number of clinical experiments with medical sponges have been done to demonstrate their efficacy. Intraoperative blood loss is minimized when gelatin sponges are used in posterior spinal fusion surgery [19]. Similarly in posterior spinal fusion surgery, clinical investigations reveal that the haemostatic collagen sponge has good haemostasis benefits, with less postoperative drainage volume and blood loss [20].

As material science advances, new approaches and technologies are applied to increase haemostatic sponge qualities. Sponge materials with haemostatic, antimicrobial, and agglutinant qualities have been designed to enhance their overall performance [21]. While there are some articles reviewing the research progress of polysaccharide-based haemostatic strategy for rapid haemostasis based on sponges, tissue adhesive, microspheres, hydrogels, cryogels and aerogels [21,22], there has been no comprehensive review of haemostatic sponges. This review article will entirely focus on sponge composite materials, their role in haemostasis and the prospects of using these sponges to stop bleeding and enhance wound closure. We will discuss the haemostatic capability of different sponges, compare their efficacy, and find out which material is the best and has the most potential. We will discuss their mechanisms to boost primary haemostatic processes, such as strengthening platelet adhesion, activation, and aggregation, as well as adsorbing fibrinogen from plasma to stimulate complement activation. We will also discuss sponge characteristics and how different sponge materials interact with and affect each component of haemostasis, as well as various approaches used to analyse their effects.

## 2 Interaction between sponge and haemostasis: underlying mechanism

Haemostatic sponges are mostly utilised in surgery, obstetrics, gynaecology, and stomatology for wound haemostasis or postoperative wound healing. As sponges have a porous structure and rapid absorption characteristics, blood on the wound surface may be swiftly absorbed by them [9]. Some absorbable haemostatic sponges on the market have considerable expansion properties (high expansion sponges). When high expansion sponges come in touch with blood, they expand quickly and effectively, compressing the wound's bleeding site. High expansion sponges are effective in clinical practice for the treatment of wound bleeding (irregular/deep/narrow) that is caused by capillaries, veins and even arterioles as they possess a high liquid absorption ratio and can absorb blood platelets easily in comparison with traditional haemostasis treatments, which involve compression, ligation, or electrical coagulation, etc [23].

Haemostasis is the process of stopping bleeding at the injury site by creating a blood clot to close the damaged blood vessels [[24], [25], [26]]. The haemostatic system consists of elements that are always present in the blood in an active state (platelets and soluble coagulation factors) that are activated immediately after an injury [27]. When blood arteries are injured, tissue factors or surface contact activates platelets, which then agglomerate to create a “platelet clog.” Prothrombin in the plasma is converted to thrombin by tissue factor III, and fibrinogen is converted to filamentous fibrin to prevent bleeding [28,29]. In general, there is a close connection between haemostasis and anti-infection. It is triggered when there is damage to the vascular wall and is made up of the intertwined activation of platelets and the coagulation cascade. This process is closely controlled by natural anticoagulants and the fibrinolytic system [30]. Blood clotting is triggered and the haemostatic system's components are directly involved in the immunological response and the immune system, which helps fight off bacterial and viral infections [31]. When there is bleeding, haemostatic materials quickly absorb the blood and generate pressure on the surrounding tissue and blood vessels. This helps to limit active bleeding and reduce the amount of time needed for coagulation, and it also aids in the healing of wounds and tends to lower levels of inflammation [32]. Despite the cessation of blood flow, the haemostatic components continue to play an important role in wound healing. Platelets and fibrin are the primary components of a blood clot, which is a complex, dynamic matrix of proteins and cells that aids in haemostasis and provides a temporary framework for invading inflammatory cells, fibroblasts, and growth factors [33]. The role of the sponge, and its haemostatic mechanisms, is discussed in detail in the following sections.

### 2.1 Effect of haemostatic sponge on RBCs, platelets and thrombin generation

The process of stopping bleeding, known as haemostasias, involves a series of steps along the coagulation pathway. Platelet aggregation producing a plug at the injured region is the primary mechanism of haemostasis. The common pathway of secondary haemostasis is comprised of the junction of the intrinsic and extrinsic coagulation pathways. Fibrinogen is transformed into fibrin via the common pathway. These fibrin subunits have a strong attraction to one another, and they link up to form fibrin strands that bind the platelets and keep the plug together. The complex method through which coagulation facilitates haemostasis by means of a cascade of clotting agents is fascinating. Overall blood coagulation involves the formation of prothrombin activator, thrombin, fibrin, and fibrinolysis [34,35].

Both the endogenous and exogenous systems are involved, from the onset of coagulation until the production of thrombin. Endogenous (or intrinsic to blood) coagulation is an independent process of blood involving different coagulation factors. The contact factor XII and factor XI are activated upon contact between the blood and the surface of the foreign body (collagen fibre of the blood vessel wall, etc.), and factor VI activates the previously dormant factor IX. Platelets can release platelet factor III by adhering to and aggregating on the surface of foreign bodies, a process called viscous metamorphosis. As soon as these active factor XI and platelet factor III enter the plasma, they react with factor VIII and calcium ions to activate the inactive factor X. In order to transform prothrombin into thrombin, factor V and platelet factor III must interact with factor X. The procedure by which fluid from the tissues enters the bloodstream is the mechanism known as exogenous (tissue origin). The active component of tissue fluid encourages the interaction between thromboplastin and plasma factor VII, which in turn activates factor X. Activation of factor X is the final step in the process. At the final stage of the process, active factor X combines with its cofactor, factor V, tissue phospholipids, platelet phospholipids, and calcium to form the prothrombinase complex, which is responsible for the conversion of prothrombin to thrombin. This thrombin cleaves circulating fibrinogen to insoluble fibrin and activates factor XIII, which covalently crosslinks fibrin polymers that are included in the platelet plug.

At the initial stage of the coagulation process, factors X and V become active, and calcium ions begin to act on prothrombin. This breaks the connection between arginine and isoleucine in prothrombin molecule, resulting in the formation of thrombin. The transformation of fibrinogen into a fibrin clot takes place as a result of the activity of thrombin. Thrombin cuts arginine-glycine link that within fibrinogen molecule, liberating fibrin peptides A and B, which results in the formation of a fibrin monomer. A fibrin polymer is formed when a fibrin monomer undergoes polymerization. The activation of factor VIII (transglutaminase) by thrombin and calcium ions, in conjunction with calcium ions, stimulates the formation of the bond between glutamine and lysine in the fibrin molecule. This procedure may result in the formation of a strong fibrin block. In addition, during the third stage of the coagulation process, the blood coagulates to create a blood cake. Nevertheless, as time passes, the blood cake becomes smaller as a result of the action of thrombus contractile proteins found in the platelets. Yet, there is a fourth stage that takes place in the body, and that is a sequence of reactions involving fibrinolysis that are produced by plasmin. These reactions are also considered as parts of the blood coagulation cascade [[36], [37], [38]].

Three distinct mechanisms—(I) red blood cell (RBC) accumulation, (II) platelet stimulation, and (III) alteration of fibrinogen structure combine to form the haemostatic mechanism.

The haemostatic system consists of elements that are always present in the blood in an active state (platelets and soluble coagulation factors) and are activated immediately after an injury The RBC enhances blood viscosity and platelet delivery to the vascular wall for the physiological haemostasis [39]. Negatively charged proteins and glycolipids are present in the RBC membrane. Electrostatic interaction between sponge and anions on the RBC surface causes intense aggregation of RBC near the wound site. Blood exposed to tissue components may interact with the platelets and activate the platelets if blood vessels are broken [40].

Endothelial cells do not adhere to platelets in normal physiological conditions. Platelet adhesion, activation, and aggregation, on the other hand, are crucial in haemostasis and wound healing. Platelets agglomerate and bind to the subendothelial matrix when vascular tissue is wounded, producing chemical signals. Active surface receptors interact with sub-endothelium and other platelets to form a strong first haemostatic block. Platelet activation, adhesion, and aggregation have all been found to be enhanced using sponges [41].

Exogenous coagulation is activated by tissue injury, but endogenous coagulation is triggered by a range of variables contained in blood vessels, resulting in a positive feedback loop that can improve the coagulation efficacy (S. [42]. The unification of these two routes results in the production of thrombin and fibrin. The conversion of soluble fibrinogen to insoluble fibrin occurs due to thrombin, so it is also considered as an important component of the coagulation cascade. It activates platelets and gives positive feedback in the process. Under the action of Ca^2+^ and thrombin, fibrinogen in the fibrin haemostatic dressing can be transformed into fibrin monomers in situ, act on the coagulation factors, and rapidly form a blood clot (X [43].

### 2.2 Effects of different sponge materials

Traditional haemostatic materials include bandages, gauzes, and sponges. In recent time, studies have been focused on the development of novel, biodegradable, and easily absorbed haemostatic gauzes or sponges. The haemostatic sponge should have moisture reservation capacity, be easy to handle, mechanically strong and reasonably priced. Furthermore, while interacting with blood, it should neither injure cells of blood nor affect protein activity [21].

#### 2.2.1 Sponges and their composite materials

**Fig. 1:**
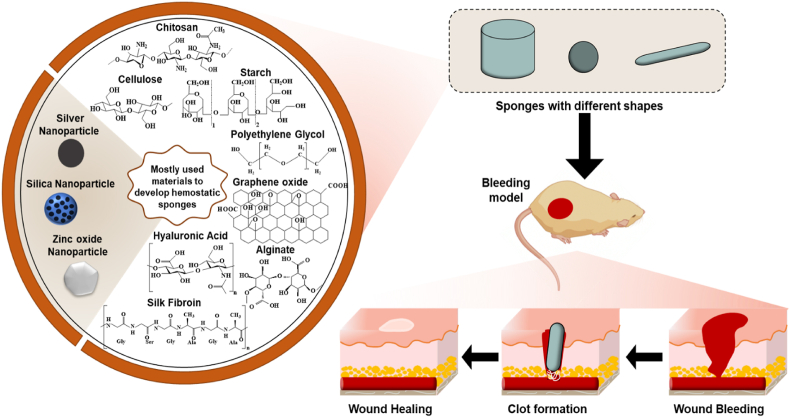
Composite materials used to form haemostatic sponges and their mechanism of action.

The porous structure of haemostatic sponges allows them to absorb a lot of blood liquid while also concentrating the coagulation components, red blood cells, and platelets. As soon as they come into contact with blood, haemostatic sponges cause platelet adhesion and aggregation, which in turn leads to platelet plug formation, preventing the flow of blood from wounds. They also trigger the release of blood coagulation-related factors involved in endogenous and exogenous coagulation pathways, resulting in stable clot formation which helps in controlling bleeding from wounds (Fig. 1) [21]. Key materials used in sponges and their working mechanisms are presented in Table 1. The development of haemostatic sponges over the last five years is outlined in Table 2.

**Table 1:** Key materials used for haemostatic sponges and their working mechanisms.

Haemostatic sponge	
Materials	Working mechanism
Chitosan	Accelerates platelet activation and adhesion by interacting electrostatically with red blood cells to accelerate RBC adherence.
Cellulose	Activates platelets, generates blood cells and water absorption.
Gelatin	Gelatin creates a physical matrix for clotting initiation by collecting water from the blood and promoting wound haemostasis. It also increases platelet adhesion, activation, and aggregation, thrombin generation, protein crosslinking to form clots, and clotting.
Starch	Absorbs liquid, accelerates the coagulation cascade.
Silk fibroin	Forms into a gel that significantly activates platelets causing platelet aggregation and adhesion and promoting blood coagulation.
Polyethylene Glycol	Creates a mechanical seal that allows blood to become trapped and solidify as a clot.
Graphene Oxide	Absorbs liquid, allows electrical and thermal stimulation.
Alginate	Activates coagulation factor XII, destroys the blood's ionization equilibrium, and absorbs fluids.
Hyaluronic Acid	Facilitates haemostasis by serotonin mediated platelet activation
**Incorporated nanoparticles**	
o Silver nanoparticles: Increase platelet activation and adhesion, exhibit an antimicrobial effect by inhibiting bacterial growth and having a wound-healing effect. oSilica nanoparticles: Advance re-epithelialization, enhance neo-angiogenesis, and enhance cell migration and proliferation (owing to moisture characteristics). oMesoporous silica nanoparticles: May promote blood clotting via an intrinsic mechanism and have antibacterial effects due to large surface negative net charges and silanol groups. oZinc oxide nanoparticles: Possess bacteriostatic properties, and they can speed up wound healing, neo-angiogenesis, and tissue integration by encouraging fibroblast adhesion.

**Table 2:** A comprehensive review of haemostatic sponges: materials, synthesis method, in-vivo models, key findings and mechanical properties.

Materials	Synthesis method	In-vivo models	Key Findings	Mechanical Properties	References
**Chitosan/cellulose composite sponge** CS: Chitosan Barca wood pulp Cel: Cellulose	Mass ratios of 9:1 were used to create the CS/Cel mixture, which resulted in the following combinations: Cel9/CS1, Cel7/CS3,Cel5/CS5,Cel3/CS7,andCel1/CS9. Freeze drying was used to get the result.	Mouse tail amputation model Rat liver trauma model Rat leg artery trauma model	The CS5/Cel5 sponge was tested in mouse tail amputation, rat liver, and rat leg artery models. This took 29, 20, and 34 s. Gauze and control groups coagulated after 3 and 4 min.	Compressive strength: 24.2 kPa Water absorption capacity: 31.21 g/g Porosity: 85.6%	[44]
**N-alkylated chitosan (AC)/graphene oxide (GO) sponge (AC/GO composite sponge): ACGS** C: Chitosan GO: Graphene Oxide	N-alkylated chitosan (AC) was produced by dissolving chitosan (CS) in acetic acid. Dodecyl aldehyde was then added. We obtained ACGS with various GO ratios (GO/AC 0%, 5%, 10%, and 20%). The names of the sponges are ACGS0, ACGS5, ACGS10, and ACGS20.	Rabbit femoral injury model	Haemostatic time for ACGS20 (GO: 20%) was (134.64 ± 17.10 s) and ACGS0 was (153.07 ± 7.33 s). The celox group had a haemostatic time of (338.29 ± 35.90 s), and the control group used conventional medical gauzes.	Porosity: 75.82 ± 1.53%, Tensile Strength: 0.11 ± 0.01 MPa Elongation: 18.88 ± 0.35% Water absorption ability (times weight): 50 Blood absorption ability (times weight): 11	[45]
**Chitosan-Tilapia Peptides Microspheres Sponge (S-CS/TPM)** CS: Chitosan TPM: Tilapia Peptides Microspheres GS: Gelatin Sponge	CS/TPM were created using ion crosslinking. Tilapia peptides (TP) were combined with Chitosan solution, then sodium tripolyphosphate was added dropwise and crosslinking was done and finally frozen and dried by lyophilization.	Rabbit ear artery haemorrhage model Rabbit femoral artery haemorrhage model	In a rabbit ear artery bleeding model, S-CS/TPM sponge had a haemostasis time of 20s, while the control group took 115s. In a rabbit femoral artery bleeding model, S-CS/TPM haemostasis took 45 s while the control group took 145 s.	Porosity: 97.39% Water absorption capacity: 3000% Platelet adhesion Rate: 72%	[46]
**Alkylated Chitosan and diatom biosilica sponge (AC-DB sponge)** AC: Alkylated Chitosan DB: diatom biosilica	First, Coscinodiscus Sp was grown in F/2 medium and diatom biosilica (DB)cells were collected. By adding an alkyl group to a chitosan molecular chain, alkylated chitosan (AC) was created. Then the mixture of AC and DB were lyophilized to form AC-DB sponge.	Rat tail transect model.	AC-DB had the shortest clotting time (106.2s) compared to the control group (595.8s).	Porosity: 85%	(X [47].
**Catechol conjugated chitosan (CHI–C) sponge.** C: Unmodified chitosan DDW: Deionized and Distilled Water	Unmodified Chitosan,CHI–C was dissolved in distilled water and placed in polythene terephthalate moulds. Moulds were filled with CHI–C or unmodified chitosan solutions which were frozen and freeze-dried.	Femoral artery bleeding model in heparinized rabbits Traumatic liver bleeding model in hemodilutional coagulopathic pigs Liver resection model in anticoagulation treated rabbits.	The CHI–C sponge stopped femoral artery bleeding in heparinized rabbits in 90 ± 0 s, faster than gauze 390 ± 60 s. In a model of hemodilutional coagulopathic pigs' traumatic liver haemorrhage, the CHI–C sponge achieved haemostasis in 3.2 ± 1.9 min, while gauze took 16.6 ± 7.2 min. Anticoagulated rabbits had similar effects in a liver resection model. Blood loss for CHI–C sponge was 9 ± 2 g, for Surgicel was 21 ± 5 g, and with TachoSil was 42 ± 45 g.	N/A	[48]
**Chitosan/Graphene oxide/Tranexamic acid composite sponge (CS/GO/TA)** Chitosan:CS Graphene oxide: GO Tranexamic Acid: TA	Ultrasound was utilised to disperse GO in water and EDC/NHS was added. After TA, the solution was freeze-dried. Lyophilized CS/GO/TA sponges were placed in (NH4)2CO3 till ammonia entered. Finally, CS/GO/TA sponges were placed in oven to remove excess NH^3^ and labelled CS/GO/TA1, CS/GO/TA2, and CS/GO/TA3.	Rat liver injury model	CS/GO/TA sponges (CS/GO/TA1, CS/GO/TA2 and CS/GO/TA3) had haemostatic times of 26, 27 and 23 s, respectively, while gauze group (control) took 109 s. On day 14, wound closure rate was over 90% whereas the control group was 75%.	Water Absorption Capability: CS/GO/TA1: 2165.00% CS/GO/TA1: 2270.05% CS/GO/TA3: 1771.42%	[49]
**Chitosan/polyvinylpyrrolidone/Zein composite haemostatic sponge (CS/PVP/Zein)** CS: Chitosan PVP: Polyvinylpyrrolidone Zein	Zein powder was dissolved in ethanol to make granules. Using distilled water and acetic acid, 10% PVP and 4% CS solutions were made. Mixtures of CS/PVP/Zein was prepared (1:0:0, 0:1:1, 1:1:0, 1:0:1, 1:1:1; 1:2:1, 1:3:1, 1:1:2, 1:1:3) and freeze-dried to make final sponges.	Rat femoral artery transection model	CS/PVP/Zein sponges exhibited reduced blood loss (1:1:1 = 3 g, 1:1:2 = 2.5 g, and 1:2:1 = 3.9 g) than gauze (control group = 4.5 g).	Swelling degree: CS/PVP/Zein sponges (1:1:1, 1:2:1, 1:1:2) reached 2100–3300%	[50]
**Micro channelled alkylated chitosan sponges (MACS)** CS: Chitosan PLA: Polylactic acid DA: Dodecyl aldehyde	The CS solution (1, 2, and 4% w/v) was dissolved in 2% acetic acid, then frozen and lyophilized. By dichloromethane leaching, microchannel CS sponges were created. PLA microfibre templates were used to make MACSs with filling ratios of 20, 40, and 60%, called MACS-1 through MACS-3.	Rat liver perforation wound model. Lethal pig liver perforation wound model	Total blood loss and haemostatic time in the rat liver model of MACS-2 (17.6 ± 4.5 g and 15s) was significantly lower than gauze (control group, 153.0 ± 15.2 gand 120s). Lethal pig puncture wound model revealed control group hemostasis in 10 min and MACS-2 in 2.0 ± 0.5 min. The total blood loss in the MACS-2 group (17.6 ± 4.5 g) was significantly lower than in the untreated group (153.0 ± 15.2 g).	Porosity: 88.8 ± 1.6% Compression Stress: 23.0 ± 1.5 kPa Water Absorption Capacity: (MACS1/MACS2/MACS3): 0.55g/cm3,1g/cm3,1.5g/cm3 Blood Absorption Capacity: (MACS1/MACS2/MACS3): 0.8g/cm3,1.2g/cm3,1.5g/cm3	[51]
**SSAD@CNC/CNF sponges (SSAD-CSs)** SSAD: skin secretion of *Andrias davidianus*, CCN: cellulose nanocrystal, CNF: cellulose nanofibers)	For SSAD-CS sponges, SSAD powder was added to CNC suspension and left overnight. After 4 h, CNF was added to the dispersion. The produced dispersion was put into a mould, frozen at 20 °C and freeze dried for 24 h, and then used to make SSAD-CS.Then haemostatic sponges with different mixing ratios were formed (SAD-CS1/SSAD-CS2/SSAD-CS3/SSAD-CS4): 6:1, 4:1, 2:1, 1:1.	Rat liver trauma model	SSAD-CS3 had less blood loss (260.6 ± 11.65 mg) than the control group (766 ± 34 mg). Blank control group had the longest bleeding time (279.6 ± 26.23 s) while SSAD-CS3 had the shortest (67.8 ± 5.07 s).	Porosity: SSAD-CS1: 99.14% SSAD-CS2: 98.91% SSAD-CS3: 98.90% SSAD-CS4: 98.79% Water Absorption Capacity: SSAD-CS1: 4500% SSAD-CS2: 4100% SSAD-CS3: 4000% SSAD-CS4: 3900%	[52]
**Chitosan/gelatin (CG) composite sponges containing oxidized cellulose fibres (OF): CG/OF** CS: Chitosan TEMPO: 2,2,6,6-tetramethylpiperidine-1-oxyl: Oxidized Fibre (OF) G: Gelatin F: Cellulose Fibres TA: Tannic Acid	Chitosan and gelatin were dissolved in acetic acid and double-distilled water. Then, CS and gelatin solutions were mixed, and deionized water was added to the cellulose suspension. Tannic acid was added to composite sponge solutions as a cross-linker. Adding 10–30% F or OF suspension to CS/gelatin makes composite sponges. After letting the solutions freeze dry, composite sponges were produced.	Rats with hepatic trauma model	Bleeding was reduced to 0.0055 ± 0.0009 g for the CG sponge, 0.0046 ± 0.0008g for 9CG/OF, and 0.0033 ± 0.0001g for 7CG/OF. Thus, the 7CG/OF sponge reduced bleeding the most.	Swelling degree: 735% ± 7	[53]
**Preparation of gelatin sponges by various crosslinking methods** Glutaralaldehyde:GTA Genipin: GP 1-ethyl-3-(3-dimethyl aminopropyl) carbodiimide:EDC n-hydroxysuccinimide:NHS Microbial transglutaminase:mTG	A 4% solution of gelatin was prepared by dissolving it in DI water at 50 °C, and then adding the crosslinkers mTG, GTA, GP, and EDC. Mixing gelatin with mTG solution at a ratio of mTG per millilitre gelatin solution yielded mTG hydrogel; adding 25% GTA solution to the gelatin solution yielded a final volume 0.5%(v/v) GTA concentration; adding GP stock solution to 4% gelatin solution yielded a final GP concentration of 15 mM; adding EDC and NHS stock solutions to 4% gelatin solution yielded final concentrations of 25 mM EDC and 10 mM NHS. In an incubator, these liquids were heated to 37° Celsius until they gelled. Hydrogels formed after 8 h of freezing at 20 °C were lyophilized for 48 h.	Subcutaneous Sprague Dawley (SD) rat model	GTA implant remaining volume was 69.1% ± 4.3%. GP–sponge and mTG–sponge residual quantities are 52.8% ± 3.5% and 27.7% ± 2.7%. EDC–sponge had the highest degradation rate, 2.7% ± 1.7%. mTG, GTA, GP, and EDC implants thickness of encapsulating tissue are 0.19 ± 0.16, 0.85 ± 0.34, 0.51 ± 0.21, and 0.59 ± 0.37 mm, respectively.	Porosity: GTA: 51.2% ± 6.1% GP: 66.6% ± 5.3% mTG: 52.9% ± 3.4% EDC: 53.5% ± 3.5% Compressive elastic modulus of wet sponge: GTA: 153.8 ± 6.9 kPa GP: 261.4 ± 13.9 kPa mTG: 67.4 ± 6.8 kPa EDC: 56.6 ± 4.2 kPa Compressive elastic modulus of dry sponge: GTA: 1088.6 ± 34.7 kPa GP: 964.2 ± 42.0 kPa mTG: 716.2 ± 94.5 kPa EDC: 714.2 ± 23.0 kPa Swelling Ratio: GTA: 956.2% ± 13.1% GP: 939.5% ± 20.7%, mTG: 1209.3% ± 57.8%, EDC: 1388.3% ± 90.9%	[54]
**Oxidized bacterial cellulose (OBC) and chitosan (CS) with collagen (COL) sponge** OBC: Oxidized bacterial cellulose CS: Chitosan COL: Collagen TEMPO: (2,2,6,6tetramethylpyperidine-1-oxyl) BNC: Bacterial Nanocellulose	The OC/COL/CS nanocomposite sponge was created by dissolving CS powder in acetic acid solution and stirring until a clear, light-yellow solution was formed. Collagen powder and lyophilized OBC were distributed in deionized water. The suspension was then agitated, and the chitosan solution was added dropwise. The resultant suspension was then freeze-dried until needed after being dialyzed with distilled water for 24 h.	Rat liver trauma model	Haemostasis time in the OBC/COL/CS group was 86 s which was the lowest of all the samples (surgical gauze 186s, OBC 175s and OBC/CS 117s). The sponge also exhibited better antibacterial properties and excellent wound healing.	Tensile strength(kPa): 128.6 ± 4.7, Young's modulus(kPa): 382.3 ± 6.7 Elongation rate (%): 4.8 ± 1.1. Swelling ratio:166%	[55]
**Thrombin loaded TEMPO-oxidized cellulose nanofiber (TOCN) -gelatin sponge (TOCN G-Th)** TOCN: TEMPO -oxidized cellulose nanofiber G: Gelatin from porcine skin Th: Thrombin from bovine plasma Bovine Plasma serum	Initially, different percentages of gelatine solution (2.5, 5, and 7.5%w/v) were created using autoclaved DI water. This TOCN suspension was then mixed with different gelatin solutions. After combining the Th solution and bovine plasma with TOCN and TOCN/gelatin, the samples were immediately frozen to prevent Th breakdown.	Rat liver avulsion model	Gelatin and Th-containing TOCN 2.5G-Th sponge stopped bleeding at the injury site the quickest (1.37 ± 0.152min), whereas TOCN sponge took the longest (4.19 ± 0.180min).	Their swelling rate was not considerably changed by TOCN 2.5G-Th.	[56]
**KR-SPs (KR12 immobilized starch-based sponge)** St: Potato starch Nor: Norbornene anhydride Peptide: Cys-KR12(CKRIVKRIKKWLR) HS-PEG-SH: Dithiol-functionalized poly (ethylene glycol) PTFE: Polytetrafluoroethylene	The first stage involved creating modified norbornene anhydride starch (St-Nor). Following lyophilization, dried ST-Nor was produced with a 90% yield by combining starch and norbornene anhydride. The final mixture was added to the PTFE moulds to create the hydrogel after this starch-based sponge (Sp) was formed by combining St-Nor with HS-PEG-SH. After lyophilization, sponges were obtained, dried, and stored for later use.	Rat liver injury model Rat femoral artery injury model	In the rat liver injury model, KR-Sp1 had a better haemostatic time (25.0 ± 5 s) than the control group (195 ± 25 s). Similar to this, KR-SP1 had a better haemostasis effect in the femoral artery injury model (95 ± 3 s) than the control group (300 ± 35 s).Thus, these KR-SPs were also regarded as an excellent antibacterial treatment for intraoperative wounds.	Compressive strength: 20–130 kPa Porosity: 91.95 ± 1.9% Maximum fluid absorption ratio (100%): 1800 ± 96%	[57]
St-PEG Sponge loaded with CMC/SA eMPs CMC: Carboxymethyl chitosan SA: Sodium Alginate St: Starch PEG: Polyethylene glycol Thr: Thrombin eMPS: electro spraying microspheres	CMC/SA eMPs were prepared using electro spraying and ionotropic gelation technology. CMC/SA eMPS were loaded into St-PEG and thrombin was also added.	Rat liver peroration wound model Rat femoral artery injury model	The haemostasis time observed in rat liver wound model was 178 ± 3.8 s in blank control group and CMC/SA eMPs showed 136 ± 2.9s and eMPs@Thr/sponge showed haemostasis in 48 ± 1.9s. Similarly, the haemostasis time observed in femoral artery injury model was 220s in control group whereas 155s and 45s in CMC/SA eMPs and eMPs@Thr/sponge.	Fluid absorption ratio: CMC/SA eMPs: 3.55 ± 0.53 eMPs@Thr/sponge: 18.10 ± 1.24	[58]
**Starch/Polyethylene glycol (PEG) sponge, Thrombin Receptor Agonist Peptide sponge (TRAP-Sp)** St: Potato Starch Nor: Norbornene anhydride TRAP-Cys-COOH: Modified thrombin-receptor-agonist-peptide	First, norbornene anhydride-modified starch (St-Nor) was created. After St-Nor solution was added to HS-PEG-SH, SDS solution was added. Deionized water was utilised to generate and wash hydrogels with varying solid contents and crosslinking levels. Sps was the lyophilization result. Similarly, TRAP-grafted Sp (TRAP-Sp) was created by immersing Sp in PI2959-containing PBS. Then, TRAP-Cys-COOH was added, and the final solution was freeze dried to obtain TRAP-Sp.	Rat liver injury model Rat femoral artery injury model.	Sp1 and Sp2 required 60±2s and 72±6s to reach haemostasis in the rat liver model. The bleeding site treated with TRAP1-Sp1 and TRAP2-Sp1 took 37 ± 3 s and 25±2s, while the control group took 228 ± 14s. While the control group achieved haemostasis in 300 ± 35s, TRAP1-Sp1 and TRAP2-Sp1 took 444 ± 3s and 33 ± 2s respectively in rat femoral artery damage model whereas Sp1 and Sp2 achieved haemostasis in 77 ± 2 s and 83± 6s.	Compressive strength of Sp1 and Sp2: 190–290 kPa Porosity: 83.4 ± 1.1% Fluid absorption ratio: Sp1: 16.5 ± 1.2 Sp2:15.7 ± 0.9 TRAP1-Sp1:16.6 ± 1.0 TRAP2-SP1:16.5 ± 0.9	[59]
**Gellable silk fibroin-polyethylene glycol sponge (SF-PEG)**	In order to achieve a mass ratio of 1/1 between SF and PEG, SF solution was combined with an equal volume of PEG 1500 solution. The combined solution was poured onto a circular dish, frozen, then lyophilized (vacuum dried).	Rabbit liver wound model	Compared to the control group 557.75 ± 42.38s the haemostatic times of the SF-PEG sponge and the gelatin sponge were 136.17 ± 62.27 s and 249.83 ± 29.18 s respectively.	Platelet adhesion rate: 84 ± 0.7%	[60]
**Fibrinogen-delivering silk sponges** SF: Silk fibroin F: Fibrinogen	The concentration ratios of silk fibroin (SF) to fibrinogen (F) in this study were 1.0% SF/2.8% F, 2.3%/1.5%, and 3.0%/0.8%. Silk and fibrinogen solutions were properly mixed and put into 48 well plates, which functioned as moulds, after being precooled to 4 °C in the refrigerator. Then freeze drying was done.	N/A	In this study fibrinogen delivering silk-based sponges was prepared. Haemostatic properties were not tested.	N/A	[61]
**Graphene-kaolin composite sponge (GKCS)** K: Kaolin powder GO: Graphene oxide	Graphene-kaolin composite sponge is prepared by mixing Graphene oxide (GO) with Kaolin powders. Following the addition of EDA, the mixture was sealed in a reaction kettle used in hydrothermal synthesis and heated to produce hydrogel. A freeze-drying process was used on the hydrogel.	Rabbit femoral artery injury model	Blood was immediately absorbed, the wound healed in roughly 73 ± 12 s, and then it was finally sewn. The operated legs regained normal motor function after one week, and there was no mortality among the rabbits.	Water absorption capacity: 706.2 ± 44.0 mg/mL Blood absorption capacity: 639.1 ± 2.5 mg/mL	[62]
**Bletilla striata polysaccharide/graphene oxide composite sponge (BGCS)** G: Graphite powder Bsp: Bletilla striata polysaccharide	Solution mixing and freeze drying were used to create BGCS (Bsp crosslinked GO composite sponge). The frozen BGCS were lyophilized after Bsp was added to the GO solution. By altering the mass ratio of Bsp to GO, the BGCS, BGCS-5, BGCS-10, and BGCS-15 were created.	Rat tail amputation model	The average bleeding duration for BGCS patients was 45.9 ± 4.6 s, however gauze did not accelerate blood clotting even after 10 min.	Porosity: 95.3 ± 0.49% Water Absorption Capacity: 5239.86 ± 129.65%	[63]
**Chitin nanofiber sponges anchored TA/CaII coating (Ch-TA/CaII)** TA: Tannic Acid CaCl_2_: Calcium Chloride Ch: Chitosan powder	A chitin nanofiber suspension is frozen to produce a sponge. The chitin nanofiber sponge turned purple when TA was introduced. Unreacted TA was rinsed from modified chitin sponges in deionized water. Steps were repeated 0 (as a control), 1, 3, and 5 times to create Ch-(TA/CaII)1, 3, and 5.	Rat Tail amputation model Rat liver laceration assay	The haemostatic efficiency of Ch-(TA/CaII)5 in rat tail amputation model was 103 ± 8.62 s and control group showed haemostasis in 265 ± 20. Similarly, the haemostatic efficiency of Ch-(TA/CaII) in rat liver laceration model was 25±3s and control group showed haemostasis in 115 ± 20s.	Compression Strength: 237.71 kPa	[64]
**ALG/HA Sponge (Alginate-Hyaluronan)** ALG: Alginate HA: Hyaluronan TA: Tranexamic Acid	Adding 10% or 20% HA to ALG solutions created ALG/HA10 and ALG/HA20. The solutions were gelled with d-gluconolactone (GDL). TA was dissolved immediately into the initial ALG/HA solution to make TA-filled sponges.The solutions were put into 24-well plates while they were still at room temperature. The ALG discs were frozen for an overnight period after they solidified, washed with deionized water, and then lyophilized.	NA	TA-loaded (ALG/HA) sponges reduced the BCI (Blood Clotting Index i.e. human blood) by 40% compared to the negative control and by 30% compared to ALG/HA20 sponges.	Porosity: 47.70 ± 3.11 Water absorption capacity (%): 5070.04 ± 114.86	[65]
**Polyvinyl alcohol/sodium alginate (PVA/SA) composite sponges** PVA:Polyvinyl Alcohol SA:Sodium Alginate GA:Glutaraldehyde	Composite sponges were made by aldol crosslinking in acidic conditions. Deionized water was used to dissolve PVA and SA. SDS solution was added. Then, glutaraldehyde was added. Freeze-drying and ultrasonic washing with deionized water generate ordered or disordered porous sponge. Microchannel S-sp is cylindrical. C-Sp1 has a single conical microchannel, while C-Sp2 has two.	Rat liver volume defect model Rat femoral artery injury model	In a liver defect model, haemostasis times of Sp and C-Sp2 were 36 ± 4 s and 31 ± 2 s. These times were shorter than the control group's (853s85±3s) and the commercial MPVA's (65±4s). The C-Sp2 femoral artery model had a lower haemostasis time (25 ± 2 s) than the control group (52 ± 4 s), commercial MPVA (47 ± 4 s), and Sp (35 ± 2 s).	Water Absorption Capacity: Sp: 2.11g/cm3 S-Sp: 2.51g/cm3 C-Sp1: 2.74g/cm3 **C-Sp2: 2.91g/cm3** MPVA: 1.34g/cm3 Blood Absorption Capacity: Sp: 1.59g/cm3 S-Sp: 1.76g/cm3 C-Sp1: 1.90g/cm3 **C-Sp2: 2.05g/cm3** MPVA: 1.45g/cm3 Compressive strength: Sp: 138 kPa S-Sp: 36 kPa C-Sp1: 86 kPa C-Sp2: 70 kPa	[66]
**Sodium Alginate/Gelatin Sponge (SA/Gel)** Gel:Gelatin SA:Sodium Alginate PHMB:Poly hexamethylene biguanide hydrochloride HA:Hyaluronic Acid	Gelatin and SA were combined, added to 12 well plates, and freeze-dried. The ionic cross-linked sodium alginate/gelatin sponge was soaked in glycerine solution after being washed with ethanol to remove unbound Ca+2 ions. After freeze drying, the sponge was labelled SA3Gel1-sLn (4,10,20,30), with n = 30 (where n is the number of bilayers).	Mouse liver injury model Infection model using mice: SA3Gel1 and SA3Gel1-sL20 were injected into S. aureus-injected mice's backs. After a week, the surrounding tissue was assessed.	SA3Gel1 sponge and SA3Gel1-sL20 blood clotting times (25.2 7.54 s and 28.8 4.08 s) were faster than commercial gelatine sponge (45±5s). The SA3Gel1 group had an apparent infection, whereas the tissue around SA3Gel1-sL20 was normal, suggesting that SA3Gel1-sL20 has remarkable anti-infectious properties and minimizes the chance of infection when bacteria infiltrate sponges.	Porosities: SA3Gel1-sL4: 90.75 ± 5.17% SA3Gel1-sL10: 76.43 ± 4.30%, **SA3Gel1-sL20: 83.17 ± 10.22%** SA3Gel1sL30:83.32 ± 8.08% Blood Absorption Rate: Commercial gelatin: 2700% SA1Gel1: 1200% **SA3Gel1-sL20: 2200%** Tensile Strength SA3Gel1-sL20: 122.9 ± 23.9 KPa	[67]
**Cross linked Hyaluronic acid/cationized dextran sponge** Dex-PDM: Cationized Dextran SHDP: HA/Dex-PDM sponge SHDQ: HA/Dex-QPDM sponge	Dextran was ionised (Dex-PDM) and then cationized (ATRP). Dex-PDM was then partially quaternized (DEX-QPDM). First, the raw material was lyophilized, then it was dialyzed and finally dissolved in DI water and HA/Dex-PDM (SHDP) and HA/Dex-QPDM (SHDQ) sponges were created.	Rat liver model	There was a notable decrease in blood loss in the SHDP-2 and SHDQ groups compared to the control group, which showed that the bleeding of the liver was successfully arrested by SHDP-2 and SHDQ (50.1 mg in the SHDP-2 group, 23.2 mg in the SHDQ group, and 200 mg in the control group, respectively).	Swelling ratio: SHDQ: 1159% SHDP-2: 1035% Porosity: SHDQ: 79% SHDP-2: 75%	[5]
**Chitin/Corn stalk/Silver Nanoparticle's sponge (CH/CS/AgNPs)** CH: Chitin CS: Corn Stalk AgNPs: Silver Nanoparticles	AgNPs were initially created using corn stalk as a reducing agent. Then, using a standard crosslinking technique, a composite sponge made of CH/CS/AgNPs was created. Chitin, cornstalk, and AgNPs solutions were combined, translated into well-plates to create a hydrogel, and then placed in a freeze dryer to form a composite sponge.	Liver injury model of rats Rat wound healing: In-vivo wound healing assessments were performed by creating two full-layer lesions on the rats' backs for adhering to the various haemostatic sponges.	CH/CS and CH/CS/AgNPs absorbed blood fast. The bleeding stopped in 31 and 30 s, while the control group took 120s.After 11 days, wound closure with the CH/CS/AgNPs sponge was 76.5% which indicate sponge's ability to heal wounds	Swelling ratio: 34% Water Absorption Ratio(g/g): 32 Saline Absorption Ratio(g/g): 27 Blood Absorption Ratio(g/G): 35 Compression Stress (MPa): 0.10 ± 0.5	[68]
**Chitosan/alginic acid/zinc oxide (CHI/AA/ZnO) nanostructured hydrogel sponges** Chi: Chitosan powder AA: AlginicAA:Algini Acid ZnO: Zno nanoparticle dispersion Genipin	CHI/AA/ZnO solutions were made by mixing chitosan, alginic acid, and different concentartions of ZnO nanoparticles. After mixing, genipin was added to each solution. Polymer solutions were placed onto an aluminium dish, frozen, then lyophilized. Same procedure for sponges without genipin (CHI) was followed.	Rabbit ear peripheral capillary haemorrhage model Antibacterial tests were performed using the sponges against *S. aureus*.	Haemostatic times were 137 ± 24.75s, 85 ± 7.07s and 93 ± 10.41s. for CHI, CHI/AA, and CHI/AA/ZnO-1. *S. aureus* growth was reduced by ZnO-infused hydrogel sponges. ZnO in the polymer network boosted antibacterial efficacy.	N/A	[69]
**MSN-GACS sponge (mesoporous silica nanoparticles-glycerol modified N-alkylated chitosan)** MSC: mesoporous silica nanoparticles G: Glycerol AC: N-alkylated chitosan	AC in acetic acid solution creates MSN-GACS. MSNs were added to the AC solution at various mass ratios (0:1, 0.25:1, 0.5:1, 0.75:1, and 1:1) and mixed using ultrasonic technology. MSN-AC suspension was lyophilized to form MSN-AC sponge.	Rat liver injury model Rabbit femoral artery injury model	In the rat liver injury model, the MSN-GACS group reached haemostasis in 64.33 ± 3.06 s, substantially faster than the CG group's 77.67 ± 4.73 s and control group's 177.33 ± 6.807 s. In the rabbit femoral artery injury model, MSN-GACS and CG groups achieved haemostasis in 69 ± 5.57 s and 71 ± 5.57 s, respectively, while the control group took 319.67 ± 31.974 s. Only the MSN-GACS group showed no damage areas after 7 days, and regenerated liver tissue resembled original tissue, indicating excellent prognosis and low cytotoxicity.	N/A	[70]
**EMQS based sponge** EW: Egg white protein QCs: Quaternized chitin derivatives MMT: Montmorillonite chitin powder EPTMAC: 2,3-epoxypropyltrimethylammonium chloride	QCs were initially made by dispersing chitin powder in KOH solution. It was done numerous times to achieve a homogeneous solution, and then EPTMAC (5,10,15) was added, and the final solution was freeze dried. Separated from the yolk, the egg white protein was forcefully agitated to generate a froth. Then MMT was stirred into the EW froth. Then EMQS (EMQS-1, EMQS-2, and EMQS-3) sponge were prepared and freeze dried.	Rat Liver injury model **Mice with a full-thickness skin defect and wound models infected with MRSA and E. coli:** The mice's backs were shaved, and 1.0 × 1.0 cm2 of their subcutaneous panniculus carnosus and full-thickness skin were taken out. The wounds were exposed to Methicillin-resistant *S. aureus* (MRSA) or *E. coli*.	In a rat liver injury model, EMQS-2 reduced blood loss from (untreated group) 163.0 ± 18.9 mg to 52.7 ± 9.1 mg and haemostasis time from 102.5 ± 13.7 s (untreated group) to 49.7 ± 8.0 s. MRSA- and E. coli-infected wounds treated with EMQS-2 healed faster. Antibacterial activity was 97.6% against *E. coli* and 98.4% against MRSA.	N/A	[71]
**Capric acid-modified chitosan (CSCA) and oxidized dextrans (ODs) sponge (C-ODs)**	CSCA was synthesized. ODS (OD1, OD2, OD3 and OD4) was prepared by dissolving DA and NaIO_4_ in PBS. Then after C-ODS was prepared by adding ODs solutions to CSCA solution. C-OD1, C-OD2, C-OD3, and C-OD4 were the final products.	Mouse tail amputation model. Rat liver injury model Rat leg artery model	In mouse tail amputation, rat liver injury, and rat leg artery injury models, C-OD2 demonstrated much faster haemostasis (325,185 and 79s) than gauze (592, 288, and 290s) and gelatin sponge (556, 285, and 215 s).	Porosity: C-OD_1_ to C-OD_4_:85.6, 85.0, 82.6, and 78.4% Fluid absorption: Each C-OD, the weight after absorption was 14–35 times its dry weigh	[72]
**OBNC-DFO sponge (oxidized bacterial nanocellulose-desferrioxamine)** BNC: Bacterial nanocellulose TEMPO: Tetramethylpiperidine nitroxide	BNC membrane was broken down into tiny bits, homogenized to generate BNC emulsion, and then freeze dried. OBNC sponge was made by resuspending BNC short fibres in sodium phosphate solution. Then TEMPO, NaClO_2_ were added into the BNC suspension. Washed with ultrapure water followed by freeze drying to produce OBNC. To make OBNC-DFO sponge, DFO was resuspended in OBNC.	Rat liver trauma model Rat tail amputation model	In rat liver trauma model, the amount of blood loss in BNC, OBNC, OBNC-DFO, COL (Collagen haemostatic sponge) was 190.47, 160.15, 159.46 and 200.26 mg mg respectively. Similarly in rat tail amputation model the amount of blood loss in BNC, OBNC, OBNC-DFO, COL was 200.42, 96.28, 80.44 and 110.37 mg respectively. Similarly, on day 21, the OBNC-DFO group's extracellular matrix remodelling, and wound healing phases are almost completed.	Tensile Strength BNC: 20.31 kPa OBNC: 40.07 kPa OBNC-DFO: 40.31 kPa COL: 248.51 kPa Youngs Modulus BNC: 1.78 kPa OBNC: 2.70 kPa OBNC-DFO: 2.73 kPa COL: 21.361 kPa Water Absorption rate BNC: 1.71 g/s OBNC: 1.81 g/s OBNC-DFO: 1.70 g/s COL: 0.11 g/s	[73]
**Collagen fiber oxidized Bletilla striata composite haemostatic sponge (CFOB)** OBSP: Oxidized Bletilla striata polysaccharide CF: Collagen fiber	The CFOB-0, CFOB-2, CFOB-6, CFOB-10, CFOB-15, and CFOB-20 were obtained by dissolving CF in acetic acid and then gradually adding OBSP to the CF solution and after that the solution was freeze dried.	Rat liver haemorrhage model	Each material had a different clotting time which was 150.4 ± 29.555 s for blank control, 73 ± 19 s for medical gauze, 50.33 ± 4.51 s for CF, and 25 ± 4.06 s for CFOB-10.	Porosity of CFOB (0,2,6,10,15,20): above 97%	[74]
**Chitin/corn stalk pith sponge (CT-CSP)** CT: Chitin powder CSP: Corn stalk pith	CT and CSP were dissolved in NaOH and combined in various volume fractions ((100% CT, 80% CT/20% CSP, 50% CT/50% CSP). Glyoxal was used as a crosslinker in the composite solution. The composite hydrogels were then created and labelled as CT100%, CT80%-CSP20%, and CT50%-CSP50%.	Rat liver bleeding model	CT50%-CSP50% sponges obtained the fastest haemostasis (70 ± 10 s), followed by CT80%-CSP20% (80 ± 10 s), CT100% (90 ± 20 s), and PVF (110 ± 15 s). After more than 3 min, the bleeding had stopped in the control group.	Compression modulus: The maximum compress modulus was found in the pure CT sponge (0.15982 MPa), followed by the pure CSP sponge (0.12376 MPa), whereas the CT50%-CSP50% sponge had the lowest (0.00334 MPa)	[75]
**Cerium-containing mesoporous bioactive glass (Ce-MBG) and chitosan (CHT) sponge**	Ce-MBG was made with different ratio of Ce and were called 2Ce-MBG, 4Ce-MBG, and 6Ce-MBG. CHT was dissolved in acetic acid and Ce-MBG was added to make Ce-MBG/CHT composite sponge. The suspension was frozen and freeze dried in 24-well plates. The names of the composite sponges are 2Ce-MBG/CHT, 4Ce-MBG/CHT, and 6Ce-MBG/CHT.	N/A	According to the in-vitro haemostatic performance haemolysis rate for the prepared Ce MBG/CHT (2.5%,1.7%,1.8%) was lower than those for pure CHT (3.01%) and GS (2.72%). The better the blood compatibility, the lower the RBC ability, and the lower the haemolysis rate.	Porosity: Pure CHT: 95% 2Ce-MBG/CHT: 90% 4Ce-MBG/CHT: 92% 6Ce-MBG/CHT: 91.5% GS: 80%	[76]
**Fenugreek gum-cellulose composite hydrogel**	Microcrystalline cellulose was agitated in Cl ionic liquid in test tubes at 100 °C for 3 h in a constant-temperature oil bath until dissolved. Fenugreek gum was added and mixed at 200 rpm/min and 100 °C for 3 h and 110 rpm/min for 2 h. To eliminate the ionic liquid in the hydrogel, an isopropanol-water solution (1:1) was added to each test tube and refilled numerous times for 14 days.	Mice liver model	The hydrogel group FCH-40% stopped the bleeding rapidly. The haemostasis time of FCH-40% was 26.33 ± 3.21 s and blood loss was 0.21 ± 0.01 g which was significantly lower than the control group (169.30 ± 9.02 s, and 0.40 ± 0.01 g].	Mechanical Strength: 28 MPa Swelling Ratio: FCH-40%: 145.7%,	[77]
**Poly-vinyl alcohol chitosan** **Sponge** CS: Chitosan PVA: Polyvinyl alcohol	CS and PVA can be crosslinked by homogeneous foaming. An exothermic reaction occurs when CS and PVA are dissolved in a weakly acidic solution and foamed under acidic catalysis. Three sponge samples were made with varied CS weight ratios (10%, 30%, and 50%). (PVA-CS10, PVA-CS30, and PVA-CS50).	Rat femoral artery puncture model Bama miniature pig femoral artery puncture model	In rat femoral artery puncture model, PVA-CS30 inhibited the bleeding within 55 s and control group (PVA) inhibited the bleeding in 85 s. In miniature pig model, PVA-CS30 stopped the bleeding in 2–3 min whereas control group (PVA) stopped the bleeding after 6 min.	Swelling Ratio PVA-CS30: 800%	[78]
**Poly (Acrylic Acid) (PAA)/Polyvinylpyrrolidone (PVP) Complex Sponge** PAA: Polyacrylic Acid PVP: Polyvinylpyrrolidone HA: Hyaluronic Acid	An ethanol solution of PAA was applied to the plastic tray and dried at 70 °C to generate a transparent layer on the bottom. Next aqueous PVP solution was added. The inflated PAA/PVP complex was cooled at −60 °C and freeze dried after standing at 25–27 °C. Before adding to the PAA film, HA was added to the PVP solution to prepare the complexes with HA.	Mice femur vein model	PAA/PVP/HA sponge stopped the bleeding within 10 min and the sponge sheet converted the blood into an adhesive hydrogel upon instantaneous absorption.	Water Absorption PAA/PVP/HA: 5.5 g/g	[79]

##### 2.2.1.1 Chitosan and cellulose based sponges

Chitosan sponge (CS) can encourage spontaneous blood agglutination, thrombin production, and the formation of new tissue for healing [9]. CS possesses excellent bio adhesion, biocompatibility, haemostatic and healing activity, bacteriostasis, and scar reduction properties [9,[80], [81], [82], [83]]. The haemostatic efficacy of chitosan sponges was investigated in rats, rabbits, and mice utilizing diverse models (liver injury model, tail amputation model, ear artery and femoral artery model). Composite chitosan-based sponges such as N-alkylated chitosan/graphene oxide porous sponge (ACGS20) stopped bleeding in 134 ± 17 s, whereas the control group (standard medical gauzes) ceased bleeding in 338.29 ± 35.90 s. Based on this study, ACGS20 is proved as a good and safe haemostatic agent [45]. Also, a composite sponge made of chitosan and cellulose (CS5/Cel5) has shown to be quite effective at stopping bleeding and speeding up the clotting process. This sponge effectively stopped the bleeding in mouse tail amputation, rat liver injury, and rat leg artery trauma in rats aged 29, 20, and 34s compared to the results generated by the gauze application (control group), which was 168 s, 172 s, and 486 s accordingly [44].

**Fig. 2:**
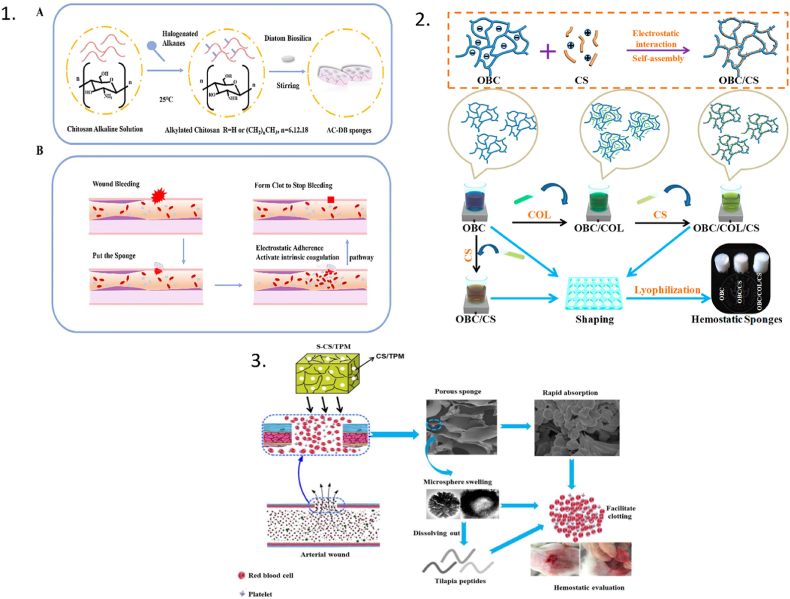
**Haemostatic chitosan sponges. (1)** Alkylated chitosan-diatom bio silica sponge. **A**). The schematic diagram representation of AC (Alkylated Chitosan) and AC-DB (Alkylated Chitosan-Diatom Bio silica sponges; **B).** The haemostatic process of AC-DB (Alkylated Chitosan-Diatom Bio silica) sponge (X [47]. **(2)** A schematic representation of the synthesis of OBC, OBC/CS, and OBC/COL/CS haemostatic sponges [55].**(3)** S-CS/TPM haemostasis (sponge containing tilapia peptides and chitosan) and its haemostatic evaluation are depicted schematically [46].

Another study by Ref. [46] demonstrated that chitosan-tilapia peptides microspheres-sponge (S-CS/TPM) is more effective than gauze at stopping bleeding. As previously indicated, the sponge took 15 s and 42 s, respectively, to stop the bleeding in the rabbit ear artery haemorrhage model and the rabbit femoral artery haemorrhage model; in contrast, the control group required 115 s and 152 s, respectively, to stop the bleeding [46]. In the same way, sponge based on alkylated chitosan and diatom-bio silica stopped bleeding in a rat tail transect model at 106.25 s, and the control group stopped bleeding at 595.8 s (X. [47], as shown in Fig. 2. This is because chitosan is a naturally occurring cationic alkaline polysaccharide. The electrostatic interaction between the anions on the surface of RBC and the positive charge of -NH^3+^ on the CS chain causes RBC to strongly aggregate around the wound site, forming blood clots that swiftly stop bleeding [39]. A catechol-cation synergy was demonstrated in one study using catechol-conjugated chitosan (CHI–C) as a haemostatic agent. The purpose of this study was to establish the haemostatic efficacy of CHI–C by forming an effective physical barrier (BpB: Blood Protein Barrier) and cellular aggregations and activations at the bleeding site using a heparinized model of femoral artery bleeding. The CHI–C sponge's haemostasis time was 90 s, which was considerably less time than that of gauze 390 ± 60 s [48]. In a pig model of traumatic coagulopathic bleeding, Kim et al. conducted a further preclinical investigation comparing a CHI–C sponge to standard haemostatic drugs before beginning clinical trials. In comparison to the gauze (16.6 ± 7.2 min), the CHI–C sponge reached haemostasis in 3.2 ± 1.9 min [48]. They underwent an ex vivo haemostasis trial on the blood of liver transplant recipients with coagulopathy. Kim et al. used a CHI–C sponge to treat coagulopathic blood from three individuals who significantly lacked haemostatic ability, and the blood coagulation was improved. The CHI–C sponge significantly improved the poor coagulation, delayed coagulation, and weak clots that were present in the blood of patient 1. In the other 2 patients, it also demonstrated improved blood coagulation [48].

Similarly, Cao et al. created the CS/GO/TA sponge by synthesizing chitosan (CS), graphene oxide (GO), and tannic acid (TA). The haemostatic times of the CS/GO and CS/GO/TA sponges were 26 s and 23 s, respectively, according to in-vivo haemostatic investigations in a rat liver damage model, while the times for the gauze group (control) were 109 s [84]. Zhang et al. conducted another experiment in which they developed chitosan/polyvinylpyrrolidone/zein (CS/PVP/Zein) sponges. When compared to the control group, an in-vivo investigation using a rat femoral artery model showed that the CS/PVP/Zein (reduced blood loss 2.4g) sponges successfully accomplished haemostasis [85]. In another study, haemostatic micro channelled alkylated chitosan sponges (MACS) were prepared by Du et al. Lethal normal/heparinized rat liver perforation wound models, normal pig liver perforation wound models, and pig femoral artery bleeding models were used to assess the haemostatic ability of MACS-2, demonstrating its promising clinical translational potential in the management of lethal noncompressible haemorrhage and promoting wound healing [51]. Additionally, CS has antibacterial capabilities. When it interacts with a bacterial cell wall, its amino group separates the wall and disrupts the cell membrane, resulting in bacterial cell lysis [86]. Considering the information shown so far, it is interesting to postulate that chitosan, which is widely acknowledged as an effective haemostasis agent, could be enhanced by being combined with other composite materials to generate even faster haemostasis.

Cellulose (Cel) is a widely used natural polymer molecule with excellent biodegradability, biological compatibility, renewable sustainability, and cost-effectiveness [87,88]. Oxidized bacterial cellulose, carboxymethylcellulose, oxidized microcrystalline cellulose, hydroxyethyl cellulose, and hydroxypropyl cellulose are all cellulose derivates with different synthesis methods, properties, and applications used in various experiments [89]. Because of its high capacity for water absorption and ability to quickly absorb blood, cellulose plays an important role in haemostasis by promoting thrombosis. Fan et al. tested chitosan (CS)/cellulose (Cel) based sponges in mouse tail amputation, rat liver damage, and rat leg artery trauma models, and discovered that haemostasis occurred in 29, 20, and 34 s for the sponges, respectively, while gauze (control) haemostasis occurred in 168s, 172s, and 486s [44]. Similarly, Yuan et al. developed a haemostatic nanocomposite by combining oxidized bacterial cellulose and chitosan with collagen (OBC/COL/CS), which demonstrated improved procoagulant and blood clotting properties, increased erythrocyte and platelet adherence, and decreased blood loss. Yuan et al. further discovered that after testing its blood clotting ability in a liver injury model in rats, bleeding stopped in just 86s, whereas surgical gauze (control group) stopped bleeding in 186s [55]. Zheng et al. developed an injectable haemostatic sponge with shape memory, high blood absorption ratio, high elastic, and antibacterial activity consisting of *Andrias davidianus* skin secretion (SSAD), cellulose nanocrystals (CNC), and cellulose nanofibers (CNFs). In a rat liver trauma model, the sponge's haemostasis time was 67.8 ± 5.07 s, which was significantly lesser than the control group (279.6 ± 26.23 s) [52]. In a rat liver model with an infected lesion caused by *Staphylococcus aureus*, the addition of carboxymethyl chitosan (CMC) and oxidized starch (OS) to a micro fibrillated cellulose (MFC)-reinforced polymer sponge improved haemostasis and wound healing. AgNPs and rhcolIII loaded sponge (A-Ag/III) showed haemostasis in 48 s whereas the control group showed haemostasis in 231s. This was due to the fast-healing effect of humanized type III collagen recombinant protein (rhCol III) and silver nanoparticles (AgNps) into the sponge. Biodegradable carboxymethyl cellulose (CMC)-Bletilla striata polysaccharide (BSP)-resveratrol (RES) sponge was developed by Chen et al. to reduce scar tissue formation after laminectomy surgery. Histological investigations confirmed that the average quantity of fibroblasts in the CMC-BSP-RES group significantly reduced following the laminectomy [90]. It was determined that the cellulose sponge's haemostatic mechanism works to speed up haemostasis by promoting platelet activation and blood absorption. It can also directly contribute to the endogenous coagulation pathway [55,90]. Similarly, there are a number of natural gums (e.g., fenugreek gum combined with cellulose) that are utilised to improve the tissue adherence of haemostatic materials, hence facilitating haemostasis. When fenugreek gum cellulose hydrogel was applied on the mice liver trauma model to analyse the haemostatic effect, it stopped bleeding in (26.33 ± 3.21) s whereas the control group stopped bleeding in (169.30 ± 9.02) s [77]*.* According to the Yuan et al., Zhen et al., Chen et al. and Deng et al. findings, cellulose sponge prepared with different derivatives are effective at adsorbing and aggregating red blood cells and platelets, which allows for the quick production of thrombus to plug the damaged blood arteries and accomplish the desired rapid haemostasis.

##### 2.2.1.2 Gelatin based sponges

**Fig. 3:**
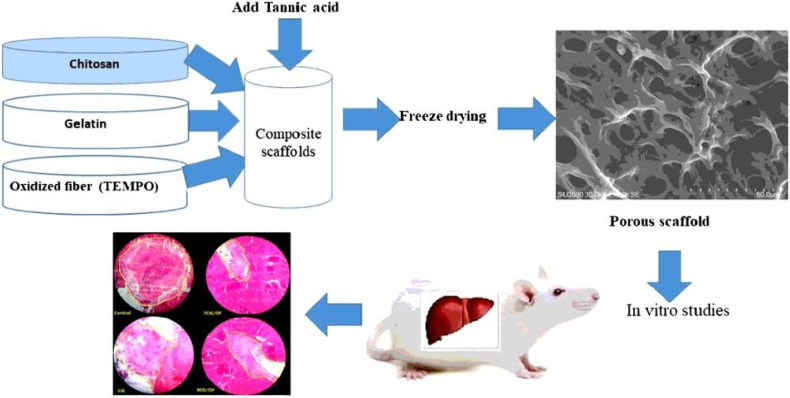
Chitosan/gelatin/oxidized cellulose sponges studied in-vitro and in-vivo as absorbable haemostatic agents according to the schematic diagram [53]**.**

Gelatin, a water-soluble protein made from animal collagen, is widely utilised as a biomaterial in food processing, cosmetics, organ-on-chip, and medicine due to its biocompatibility, biodegradability, and biosafety [[91], [92], [93], [94]]. When applied to a wound, gelatin promotes clotting by increasing platelet adhesion, aggregation and activation as well as thrombin generation, protein crosslinking, and haemostasis. A gelatin sponge can stop bleeding by absorbing water from the blood and making it sticky [95]. Ranjbar et al. made CS/gelatin composite sponges with oxidized cellulose fibres. Oxidized cellulose fibres enhanced the sponge's swelling capacity [53] as shown in Fig. 3. The sponges were tested in vivo on a liver-damaged rat model. OF (oxidized fibres) enhanced the clotting ability of CS/gelatin sponges. Gelatin's (G) negative charge can also activate coagulation factors, increasing fibrin and platelet production. After absorbing water, the gelatin sponge swells, providing mechanical compression and adhesion to the bleeding wound surface, promoting blood coagulation and wound healing. The amount of bleeding in the control group was 6 ± 1.5 mg, but in the 7CG/OF sponge group, the amount of bleeding was reduced to 3.3 ± 0.1 mg [95]. Similarly, Ibne et al. prepared (2,2,6,6-Tetramethylpiperidin-1-yl) oxyl) TEMPO-oxidized nanocellulose (TOCN)/biopolymer gelatin (G) sponges. An in-vivo experiment was performed in a rat liver avulsion model, and the bleeding period was reduced by the addition of thrombin (Th) and gelatin [56]. The absorbed blood volume of the TOCN 2.5 G-Th sponge was greater, and the bleeding time was 1.37 ± 0.152 min, which was less than that of the TOCN sponge, which had the longest bleeding period (4.19 ± 0.180 min). Thrombin, a naturally occurring polymer with many positive and negative functional moieties ideal for crosslinking processes, may be efficiently linked to gelatin, making gelatin a good polysaccharide. It also serves as a physical matrix for the initiation of clotting by absorbing water from the blood and making it sticky. Additionally, gelatin's negative charge has the ability to activate several coagulation factors, accelerating the production of fibrin and platelet aggregation [95].

Gelatin sponges swell when exposed to water, exerting mechanical compression, or filling the wound site. This enhances blood coagulation and aids in wound healing. Due to their low mechanical strength and high susceptibility to hydrolysis, gelatin sponges are made using a variety of crosslinking agents. Therefore, gelatin sponges are stabilised through the use of material crosslinking with substances like glutaraldehyde (GTA), genipin (GP), 1-ethyl-3-(3-dimethyl aminopropyl) carbodiimide (EDC), and microbial transglutaminase (mTG) to boost their strength and hydrolysis resistance and keep them stable. Gelatin sponges were implanted in subcutaneous pockets on their dorsal surfaces of adult SD rats for biocompatibility test. The implanted glutaraldehyde (GTA) one demonstrated the lowest degradation rate, with a persisting volume of 69.1% ± 4.3%, despite the fact that the implanted samples were of varying sizes and shapes. The degradation rate was highest for the EDC sponge, with a residual volume of just 2.7% ± 1.7% compared to 52.8% ± 3.5% and 27.7% ± 2.7% for genipin (GP) and mTG sponge, respectively. Additionally, the average thickness of encapsulating tissue around mTG and EDC implants showed 0.19 ± 0.16 and 0.59 ± 0.37 mm whereas GTA and GP implants showed 0.85 ± 0.34, 0.51 ± 0.21 respectively. Thus, the mTG-crosslinked gelatin sponge is a promising material for use in the tissue engineering [54,96,97]. Since GTA is able to successfully stabilise collagen or its derivatives, it is widely used in biomedicine, but it also shows considerable cytotoxicity due to the difficulty in eliminating the remaining aldehyde, which may cause damage to the cells, whereas EDC degrades rapidly. After subcutaneous implantation, the GP material is hard and shows severe tissue rejection, whereas the mTG-sponge demonstrates the most overall performance due to its high porosity, mechanical strength, and high resistance to degradation [98].

Generally, gelatin when combined with other composite materials (chitosan, oxidized fibres, TEMPO-oxidized nanocellulose) promoted blood coagulation, rapid absorption and wound healing. When gelatin sponge was prepared alone, the incorporation of crosslinking agents could improve the sponge's characteristics (i.e., mechanical strength) and maintain haemostasis.

##### iii Starch-based sponges

**Fig. 4:**
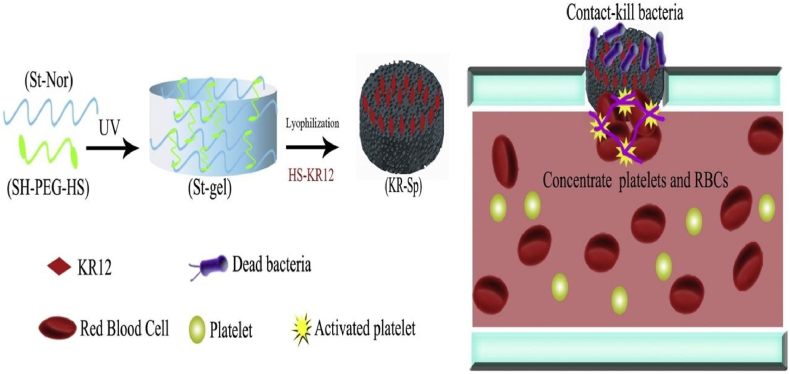
The schematic diagrammatic representation of starch-based macroporous sponges (KR-Sps) covalently labelled with the antimicrobial peptide KR12 via the highly efficient thiolene photo click (SH-PEG-HS; dithiol-functionalized poly (ethylene glycol), St-gel: Potato starch gel, KR-Sp: KR12 immobilized starch-based sponge) [57]**.**

A naturally biodegradable, inexpensive, water-soluble, and biocompatible substance is starch. The body's enzymes may also quickly break down starch into oligosaccharides, maltose, and glucose, which can then be absorbed by the tissue [99]. The antimicrobial peptide KR12 was covalently bonded to starch-based macroporous sponges (KR-Sps) using a highly effective thiol-ene photo click reaction. In an in-vivo experiment, the KR12 immobilized starch-based sponge (KR-Sp) performed better as a rapid haemostatic material. In rat liver injury and femoral artery models, the sponge exhibited haemostasis times of 25.0 ± 5 s and 95 ± 3 s respectively while the control group showed haemostasis time in 195 ± 25 s and 300 ± 35 s, respectively. Similar to this, methicillin-resistant *Staphylococcus aureus* (MRSA) and Gram (+) and (-) bacteria were resistant to the inherent antimicrobial capabilities of KR12 immobilized sponges for at least 5 days. These findings support the use of KR12 peptide-immobilized starch sponges for haemorrhage control and antimicrobial activity [57]. Due to its well-known antibacterial action and relatively simple synthesis procedure, KR12 (KRIVKRIKKWLR) was utilised as an antimicrobial peptide. KR-Sp absorbed blood fluids and concentrated platelets and RBCs at the bleeding site, according to adhesion tests. Starch absorbs liquid and speeds up the natural coagulation cascade, in which platelets aid in the formation of the main haemostatic block and RBCs aid in the formation of the ultimate thrombus [23] as shown in Fig. 4 [57]. Similar research was conducted by Liu et al., who sprayed carboxymethyl chitosan and sodium alginate electrosprayed microspheres (CMC/SA eMPs) onto a starch-polyethylene glycol sponge (St-PEG sponge) for topical administration in order to create a biocompatible and effective haemostatic substance. The liver perforation wound in rats was selected as a typical model to assess the haemostasis ability of prepared haemostatic materials in-vivo. In this model, haemostatic time of eMPs@Thr/sponge(CMC/SA eMPs encapsulating thrombin compounds) was shortest (48 ± 1.9s) among all other groups, which proved that the eMPs@Thr/sponge could combine the qualities from the eMPs/sponge and thrombin and demonstrated outstanding haemostatic performance [58].

##### 2.2.1.4 Silk fibroin and poly (ethylene glycol) based sponges

Silk fibroin (SF) is a naturally occurring polymeric fibrin that is generated from silk. SF possesses excellent mechanical, biocompatibility, and biodegradability characteristics (W [100]. It has been demonstrated in a large number of research that soluble SF sponge could induce blood coagulation rapidly [101]. In the pharmaceutical industry, polyethylene glycol (PEG) is used as an excipient to control the viscosity and melting point of medication formulations. PEG has a low vapour pressure and excellent thermal stability, and it is solvable in water, ethanol, and a variety of other organic solvents. It is non-toxic and can be used for surface coating, sponge manufacturing, capsule manufacturing, and plasticization. SF-PEG sponges (sponge based on silk fibroin and PEG) that could be solubilised and transformed into a gel by blood were created by mixing SF and PEG (PEG, 1500Da) and lyophilising the mixed solution. The SF-PEG sponge, along with a control sample (SF sponge (no PEG)) and a commercial gelatin sponge, were tested for haemostasis using a rabbit liver trauma model, resulting in bleeding times of 136.17 ± 62.27 s in the SF-PEG sponge group, 557.75 ± 42.38 s in the control group, and 249.83 ± 29.18 s in the commercial group (gelatin sponge) [60]**.** The haemostasis in this study was significantly influenced by SF. The bleeding port was first physically stopped by the SF gel that was created when PEG was present. The biological process of blood coagulation may also be affected by SF, which brings us to our second point. SF can activate platelets in-vitro, stimulate platelet aggregation and adhesion, maintain platelet activity, and stimulate blood coagulation, while PEG competes for water after the sponge creates a mechanical seal under which blood clots. Silk protein sponges were created by Teuschl et al. by combining the coagulants with an aqueous silk solution, moulding, freeze-drying, and water annealing the mixture. The haemostatic potential of fibrinogen and/or thrombin was successfully maintained while being transported through silk. In the current study, fibrinogen was liberated from silk sponges while remaining connected to the spongy silk structure. As a result, the silk-based carrier system and the fibrin network provided by the sponge for in-vivo applications were not separated, thereby supporting the overall haemostatic device's anchoring features [61].

In general, SF when employed alone was not as effective as employed in combination with PEG. The incorporation of fibrinogen and thrombin in SF sponges developed by Teuschl et al. might improve the haemostatic effect, however, it was not tested and reported in that study.

##### 2.2.1.5 Graphene oxide-based sponges

Due to its hydrophilicity, electronegativity, and adaptability, the two-dimensional carbon nanomaterial known as graphene oxide (GO) has attracted considerable interest [102]. Quan et al. developed a cross-linked graphene sponge (CGS) that is an excellent haemostat. The haemostatic effectiveness of the cross-linked graphene oxide was evaluated using a tail amputation model in rats, and gauze sponge was utilised as a control [103]. Cross-linked graphene oxide sponge was able to control the bleeding within 240 s, while the gauze group (control) did not stop the bleeding even after 10 min [103]. It has been demonstrated through a variety of studies that materials based on graphene oxide offer notable benefits, such as large pore volumes, high surface areas, and excellent structural stability, which makes it appropriate for use in haemostatic applications [[104], [105], [106]]. Graphene that has been cross-linked also exhibits a remarkable capacity for rapidly absorbing liquid, which serves to enhance the concentration of blood cells and speeds up the process of blood clotting [107]. Moreover, Wenjing et al. developed gelatin (GP) and graphene oxide (GO) accounted for 0,1,5,10, and 20 wt% of the total solid mass, and composite sponge (GP-GO) was created donated as GP, GP-GO_1_, GP-GO_5_, GP-GO_10_, and GP-GO_20_. These samples exhibited stable cross-linked and outstanding absorbability [108]. The haemostatic impact of the GP-GO sponge with 5% GO was tested using two distinct rat models, including the puncture of the femoral artery and the puncture of the liver. In femoral artery puncture model, it was capable of stopping the bleeding in 77 ± 17 s, which was a much shorter than the GP and Celox™ sponges (117 ± 11 s and 101 ± 9 s, respectively). In rat liver puncture model, it stopped bleeding in 36.0 ± 5.3 s. These findings demonstrated GP-GO potential for the treatment of noncompressible wound haemorrhage. The incorporation of GO improved the structure of the sponge and altered its degree of wettability [108].

**Fig. 5:**
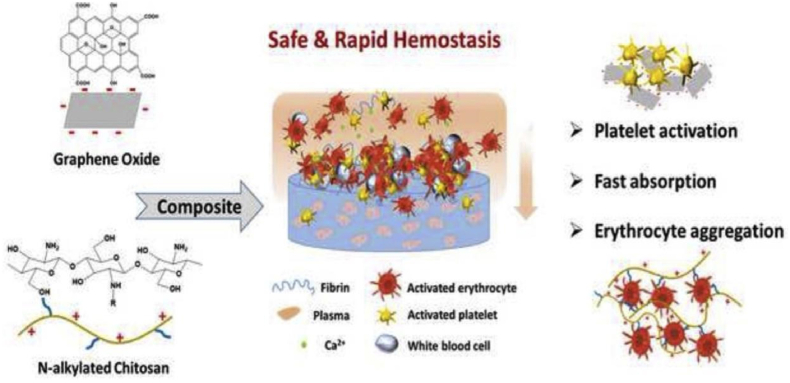
The ACGS20 (N-alkylated chitosan/graphene oxide porous sponge with 20% ratio) haemostasis mechanism is depicted in a schematic diagram [45].

Similarly, graphene oxide (GO) and N-alkylated chitosan (AC) sponges are intriguing options for an emergency haemostat. However, GO sponges may cause toxicity, and AC sponges have poor mechanical strength. As illustrated in Fig. 5, a series of GO/AC composite sponges (ACGS) were created with different ratios (GO/AC, 0%, 5%, 10%, and 20%) to address these issues. The control groups were sterilized Celox, GO, and regular medical gauzes, while the treatment groups were sterilized ACGS0 and ACGS20. The haemostatic time of ACGS20 was about 134.64 ± 17.10 s, ACGS0 was 153.07 ± 7.33 s, Celox was 164.52 ± 20.99 s, GO was 181.25 ± 19.37 s, and the haemostatic time of standard medical gauze was 338.29 ± 35.90 s. In a rabbit femoral injury model, N-alkylated chitosan/graphene oxide porous sponges with a 20% GO ratio (ACGS20) demonstrated the best blood clotting effect [45]. Thus, GO not only increased AC's mechanical strength and liquid adsorption capabilities, but it also raised platelet activation and encouraged intracellular Ca^2+^ release. This is because GO has a strong affinity for water and sponges made of graphene can quickly absorb liquid due to their porous nature. As a result, the first step in the haemostasis process, the rapid absorption of plasma and enrichment of blood cells, can be achieved by using GO-based haemostatic materials. In addition, GO is easily multifunctionalised and can be effortlessly incorporated with other coagulation molecules. All these multiple factors resulted in the ultrafast haemostasis effect of graphene-based haemostatic sponges [45]. Chen et al. created a novel material called the Bletilla striata polysaccharide/graphene oxide composite sponge (BGCS) [63]. BGCS could stop bleeding in a rat tail amputation model in 50 s by promoting strong platelet stimulation and red blood cell aggregation, as well as accelerating fibrin formation and blood coagulation [63]. Thus, graphene aerogels in GO were capable of rapidly absorbing blood plasma, letting blood cells to assemble on the surface and encouraging blood coagulation on the wound surface [45].

Graphene-based sponges, in conclusion, have a high rate of fluid absorption, which allows them to quickly absorb blood from wounds, activate clotting pathways, and accomplish rapid haemostasis. Moreover, graphene oxide also enhances the sponge's mechanical strength.

##### 2.2.1.6 Alginate based sponges

Given that pure alginate (ALG) has high water solubility but poor mechanical strength, it must be modified to broaden its scope of use [109]. Alginate is biocompatible, biodegradable, has a good water absorption ratio, and is non-toxic [110]. Large amounts of wound exudate can be absorbed by alginate-based sponges, which also offer a physiologically moist microenvironment to aid in wound healing. Alginate sponges, meanwhile, help lessen discomfort and stop additional bleeding of the wound because of their superior gelation capacity [111,112]. Catanzano et al. developed tranexamic acid (TA) loaded alginate and hyaluronic acid sponge using an easy internal gelation procedure followed by a freeze-drying step which showed that among the different concentration ratios of ALG/HA, ALG/HA20 sponges reduced blood clotting by 30%. This sponge was prepared mainly to reduce the tooth extraction bleeding and risk of alveolar osteitis [65]. Because of its many useful properties, including its great absorbency, its capacity to expedite healing and epidermal regeneration, and its activation of platelets and blood coagulation, ALG has become the most common wound dressing material. In addition, HA and TA also plays a vital role in this composite sponge as HA is present in both mineralized and nonmineralized tissues of the periodontium, it promotes the regeneration of both the soft and hard tissues involved in periodontal health [113]. TA was commonly administered as a mouthwash or as socket irrigation immediately after extraction to stop post-extraction bleeding in patients on warfarin [114]. Che et al.'s sodium alginate/gelatin sponge (SA/Gel) demonstrated antibacterial activity as well as synergistic haemostasis. Sponges with different sodium alginate/gelatin ratios, SA3Gel1 and SA3Gel1-sL20 demonstrated faster haemostasis 25.2 ± 7.54 s, and 28.8 ± 4.09s respectively as compared to the commercial gelatin sponge (45.6 ± 1.57s) in liver damage model of mouse. Similarly, results also proved that, bacterial colonies reduced after injecting these sponges on to the back of *S.* aureus injected mice [67]. The haemostatic characteristics of the sponge were affected by its carboxy group content and the amount of calcium ion it contains whereas because of the porous nature of the sponge, the blood cells were able to physically penetrate the sponge, cluster together and eventually formed as clot as the blood was absorbed. Along with that, alginate surfaces have a negative charge, which may speed up the clotting process by activating coagulation factor XII and the addition of crosslinking agent CaCl_2_ also had the ability to further increase the coagulation potential by contributing to both endogenous and external haemostasis [115].

Generally, alginate is biocompatible, biodegradable, has high water solubility but poor mechanical strength. Therefore, alginate has not been used alone, but incorporated with other agents to achieve effective haemostatic sponges. The porous structure of the alginate-based sponges makes it possible for them to rapidly absorb a significant quantity of water, concentrate plasma, promote the accumulation of endogenous coagulation components in the wounded area to stop bleeding, and heal the wound.

##### 2.2.1.7 Hyaluronic acid-based sponges

**Fig. 6:**
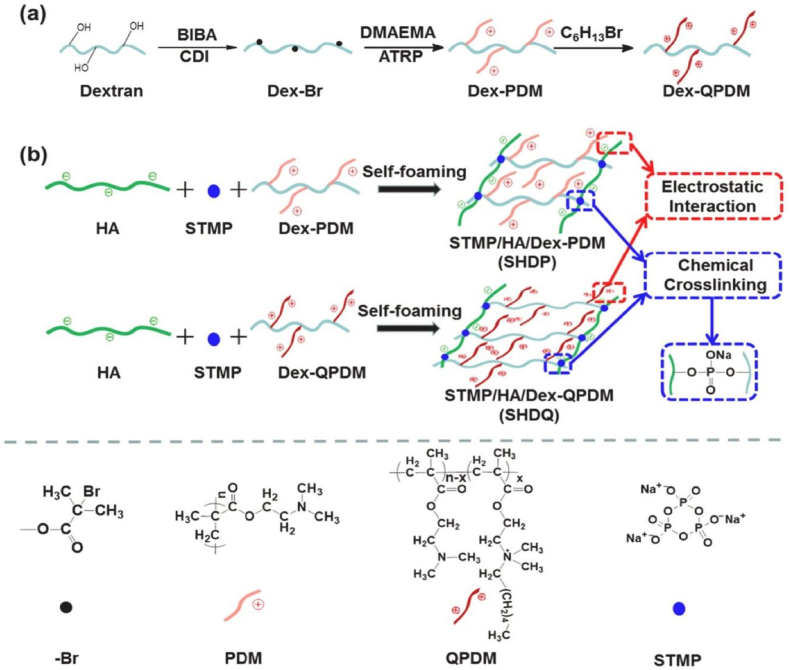
Schematic Diagram of the preparation of **(a)** cationized dextran (poly (2-dimethyl amino)-ethyl methacrylate)-grafted dextran (Dex-PDM)) and **(b)** haemostatic sponges [5].

In the realm of biomedical materials, hyaluronic acid (HA) is a natural polysaccharide that is biocompatible, biodegradable, and bioabsorbable [116]. The improved grafted HA possesses quick water absorption, strong biocompatibility, and certain adhesion properties [117]. As a result, HA can be converted into a spongy state for usage as haemostatic material. Because of its ability to compress the wound surface and stop bleeding via physical channels, it can absorb wound exudate, increase the concentration of clotting components in the blood, and speed up coagulation. This can reduce blood loss and reduce the time needed for coagulation [118]. Using a straightforward self-foaming procedure, Liu et al. produced a polysaccharide-based haemostatic porous sponge from cationic dextran (Dex-PDM) (poly((2-dimethyl amino)-ethyl methacrylate)-grafted dextran) and hyaluronic acid (HA) [5] as shown in Fig. 6. In-vivo haemostatic performance of SHDP and SHDQ sponges were evaluated in a rat model of haemorrhaging livers. After inducing bleeding, the pre-weighted SHDP or SHDQ was placed on the bleeding site with tweezers. The blood-soaked sponge was weighed after 60 s. SHDQ stopped liver bleeding in 1 min with a reduction in blood loss of 23.2 mg, whereas SHDP reduced blood loss by 50.1 mg. As a result, SHDQ demonstrated excellent in-vivo haemostatic performance and has the potential to be a promising haemostatic sponge for haemorrhaging tissues or organs [5].

##### 2.2.1.8 Sponges based on combination of natural and synthetic polymers

Because of their beneficial properties, such as biocompatibility, bioactivity, degradability, suitable viscoelasticity, and ease of processing, natural polymers have found widespread applications in biomedicine, including for haemostasis and wound healing. Chitin, chitosan, alginates, and hyaluronic acid are some examples of natural polymers [119]. A description of sponges made from these polymers can be found in section 2.2 (I). To enhance haemostatic properties, natural polymers have been combined with synthetic polymers to prepare sponges. For instance, fibroin, a silk protein, does not have haemostatic potential because it lacks functional groups that encourage clotting. Porous films made from electrospinning silk fibroin, on the other hand, can be co-processed with other synthetic (poly vinyl alcohol, poly (l-lactide)), or natural polymers (gelatin, collagen, chitosan) to act as haemostatic materials [120,121].

Due to their ability to overcome the disadvantages associated with natural polymers, synthetic polymers are finding increasing applications in the field of haemostasis. Because of their strong adhesion to tissue and histocompatibility, materials based on modified polythene glycol (PEG) are frequently utilised as physical barriers. This is because these materials have a high level of tissue adhesion and are also good carriers for therapeutic medicines [122]. In addition, they have high water-absorption characteristics, which concentrates coagulation factors and platelets, boosting platelet aggregation. This is important since platelets are responsible for primary haemostasis [123]. To make synthetic poly-vinyl alcohol-chitosan sponge (PVA-CS), Zhao et al. cross-linked PVA and CS while the mixture was being foamed and then cross-linked. The percentage of CS in each of the three sponge samples that were made was 10%, 30%, and 50% respectively (PVA–CS10, PVA–CS30, and PVA–CS50). The PVA–CS30 groups produced better results than the PVA and gauze groups in the rat femoral artery puncture model. These groups also showed the fastest haemostasis, as the bleeding was stopped within 10 s. In the same manner, the PVA-CS30 sponge used in the miniature pig model stopped the bleeding in two to 3 min, and rebleeding did not occur when the materials were removed, but the gauze group was unable to stop the bleeding [78]. PVA-CS30 sponge, in particular, has been shown to have excellent haemostatic effects amongst those made from CS in a variety of weight ratios. It's because of PVA and CS characteristics as PVA does not have the function of haemostasis on its own, but it does generate physical haemostasis by local compression; CS is a potent natural haemostatic; and because of its positive charge, CS attracts red blood cells and platelets to promote thrombosis [124].

In addition, previous research has also shown that the PVA-CS sponge's irregular porous structure encourages red blood cell agglutination, platelet activity, and thrombin synthesis, all of which work together to produce a rapid haemostatic [125]. In a similar manner, Ito et al. fabricated a sponge composed of poly (acrylic acid) (PAA), polyvinyl pyrrolidone (PVP), and hyaluronic acid (HA). Haemostatic effect was assessed on femur vein of mice model where PAA/PVA/HA sponge (7mm × 7 mm × 2.5 mm) was placed on the bleeding region. Within 10 min, the PAA/PVP/HA sponge sheet converted the blood into an adhesive hydrogel upon instantaneous absorption, adhered firmly to the haemorrhage site, and successfully stopped the bleeding [79,126]. In addition, a clinical investigation on haemostasis after blood dialysis was carried out utilizing a PAA/PVP/HA sponge sheet that had a cotton liner. Following the completion of the dialysis procedure, a sponge sheet was placed over the skin surface around the injection site. Once the needle was taken out, a light pressure was applied to the sponge sheet for a period of 60 s. During the treatment, there was no evidence of blood leaking through the sponge sheet. After that, the sponge was removed carefully, and it was determined that complete haemostasis had been achieved [79]. Also, haemostasis following tooth extraction was also explored using the PAA/PVP/HA sponge in stick form (7 mm × 7 mm × 25 mm). The complicated stick placed into the socket quickly expanded, and the hydrogel attached to the bleeding tissue, successfully stopped the haemorrhage. However, this trial was performed in a small number of patients (11), and PAA/PVP/HA sponge did not show statistically significant efficacy when compared with PA/PVP sponge but they did show the considerable potential of the PAA/PV/HA complex sponges as novel haemostatic materials [79]. The bio adhesive PAA/PVA/HA sponge is made up of low-toxic polymers and offers a substantial amount of unexplored potential for future applications. PAA has a high solubility in water, and when it is combined with water, it physically binds to PVP to produce a complex that is insoluble in water. The formation of hydrogen bonds between the carboxyl groups from the side chains of PAA and the pyrrolidinyl pendant groups from PVP is one of the most important interactions that take place between PAA and PVP. Hence, when the PAA/PVP complex gel is subjected to physiological circumstances, the carboxyl groups of PAA are gradually neutralised by the penetrating cations, which results in the dissociation of the complex into the original water-soluble polymers [126,127].

In conclusion, while natural polymers have numerous uses in biomedicine, especially in the areas of blood coagulation and wound repair, sponges and hydrogels have been produced by combining natural polymers (such as cellulose, chitosan, and gelatin) with synthetic polymers (polyvinyl alcohol, polyethene glycol) for improved haemostasis. The results of these studies demonstrate that synthetic polymers are increasingly being used in the field of haemostasis due to their high haemostatic efficacy, low toxicity, and excellent tissue adherence.

##### 2.2.1.9 Other composite materials-based sponges

There are plenty of other materials which are used to prepare haemostatic sponges. Egg white protein (EW) is a key component of EMQs sponges, which also include quaternized chitin derivatives (QCs), which have been shown to be a broad-spectrum antibacterial polymer even to some drug-resistant bacteria, and montmorillonite (MMT), which helped to improve mechanical properties. These characteristics accounted for the superior haemostatic performance shown in the rat liver injury model ((49.7 ± 8.0 s) compared to the control group (102.5 ± 13.7 s) [71]. The C-ODs sponge was made by modifying dextran (DX) using a normal oxidation approach to create oxidized dextran and then synthesizing CSCS by acylation by grafting capric acid (CA) partially onto the C2 position of CS (chitosan). Three injury models (mouse tail amputation model, rat liver injury model, and rat leg artery injury model) were used to determine the haemostatic effect; results showed that C-OD2 (325 s, 85 s, and 79 s) were significantly more effective at stopping bleeding than the control group (592 s, 288 s, and 290 s) and commercial gelatin sponges (556, 285, and 215 s). As a result, the C-ODs sponge was able to stop bleeding because of the superior absorption, tissue adherence, and haemostatic activity of the CS and OD [72]. Similarly, OBNC-DFO haemostatic sponge was prepared by Bian et al. which comprises of oxidized bacterial nanocellulose (OBNC) and Desferrioxamine (DFO). The haemostatic ability of OBNC-DFO, OBNC, COL, and BNC sponges was studied in rat tail amputation and liver trauma models which showed that blood loss was 190.47, 160.15, 159.46, 200.26 mg and 200.42, 96.28, 80.44,110.37 mg respectively [73]. Thus, the high porosity and absorption capacity of the OBNC-DFO sponge make it a promising material with potential for activating coagulation pathways. A haemostasis test was performed on a rat liver haemorrhage model, and the results showed that CFOB-10 (10% Oxidized Bletilla Striata Polysaccharide; OBSP) had a clotting time of 25 ± 4.06 s, while the clotting time for the control showed 150.4 ± 29.555s, demonstrating that CFOB had good haemostatic characteristics [74]. Chitin (CT)-Corn Stalk Pith (CSP) sponge prepared by Chen et al. demonstrated that CT50%-CSP50% sponge achieved haemostasis in around 70s in a liver injury model in rat which was quickest as control group took 3 min to stop bleeding [75]. CSP can absorb Ca ions, which contribute to the regulation of coagulation, which is imperative in the maintenance of haemostasis. The haemostatic effect was due to the procedure as CSP was integrated into chitin to reduce the crystallinity and enhance the softness of the chitin sponge and CSP also improved the softness of the chitin sponge. Using the freeze-drying technique, Liu et al. was able to develop haemostatic sponge (Ce-MBG/CHT) from chitosan (CHT) and cerium-containing mesoporous bioactive glass (Ce-MBG). When tested with *E. coli*, it was found that the antibacterial activity of Ce-MBG/CHT sponges decreased with increasing Ce doping, with 6Ce-MBG/CHT having the weakest (50.48%). Comparatively, in the presence of *S. aureus*, 4Ce-MBG/CHT demonstrated a greater antibacterial activity (93.36%) than other groups. The in-vitro haemostatic performance of composite sponge materials was evaluated by comparing them with commercially available gelatine sponge (GS), showing superior antibacterial activity against *E. coli* and *S. aureus* and providing rapid and effective haemostatic treatment when tested with citrated human blood [76]. However, more study is needed to verify this effect in vivo and determine the haemostatic mechanism underlying the enhanced haemostatic impact [75,76,128]. Similarly, oxidized dextran can be crosslinked with amino-containing crosslinking agents via aldehyde groups from oxidized dextran in order to generate blood-absorbing sponges and hydrogels [129,130]. Furthermore, the aldehyde groups can crosslink with the amino groups in tissue, leading to improved haemostasis through enhanced tissue adhesion. In order to create effective haemostatic materials for wound healing, it is crucial to design molecules containing aldehyde groups.

##### 2.2.1.10 Commercial sponges

**Fig. 7:**
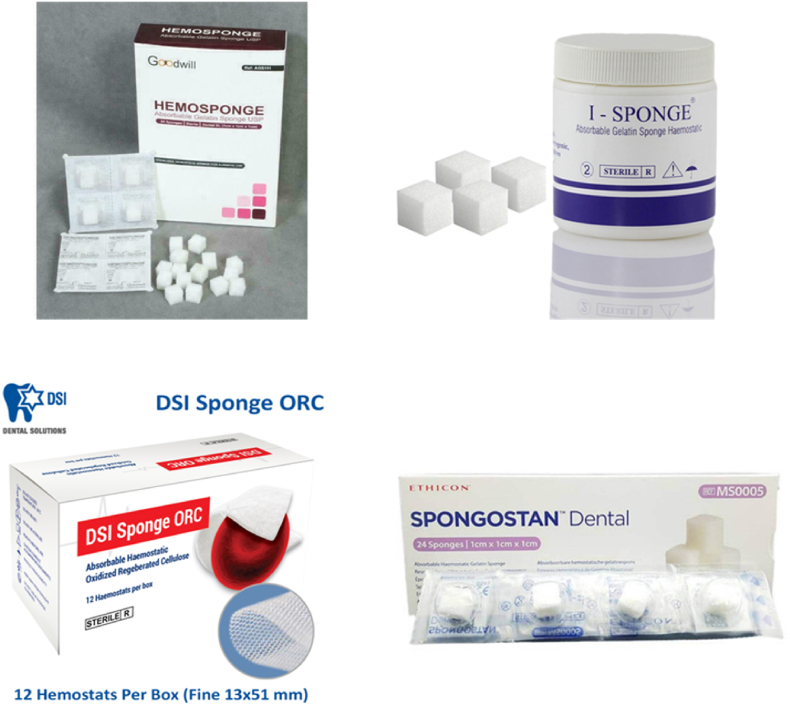
Available commercial sponges on the market.

The market is stocked with a wide variety of commercial sponges in a variety of shapes and sizes. The following are some examples of commercial sponges: goodwill hemosponge dental absorbable gelatin sponge USP (10 mm × 10 mm × 10 mm) (Goodwill:ebay.com.au/itm/115149039777), spongostan absorbable haemostatic gelatin sponge (1 cm × 1 cm × 1 cm) (Ethicon; ahpdentalmedical. com.au), DSI ORC dental surgical sterile absorbable haemostatic sponge (13 × 51 mm) (DSI: ebay. com.au/itm/164510323439) and I-sponge absorbable gelatin sponge (My Dent Store:ebay.com.au/itm/151168764444) which are illustrated in Fig. 7.These information are taken from their website. The companies claimed that when placed at the injury sites, these sponges, which are made up of a porous network, have the ability to absorb bodily fluids. DSI ORC sponges and I-sponge are biocompatible, non-toxic, non-allergenic, non-immunogenic, and non-pyrogenic, according to their websites. Moreover, they are claimed to not produce any immune reactions. These sponges are used in surgical procedures to prevent bleeding from capillaries, veins, and smaller arteries. When ligation and other traditional means of establishing haemostasis are either unable to stop the bleeding or pose a significant risk, medical professionals turn to the usage of commercial sponges like these. Scissors that have been properly sterilized can be used to cut the material. It should be administered directly under pressure to the surface of the wound where the bleeding is taking place. DSI ORC is absorbable haemostatic oxidized regenerated cellulose, which changes the structure of albumin and blood globulin, and contributes to the onset of haemostasis. DSI ORC sponges produce an area with a low pH, which results in localised vasoconstriction. The natural ingredient in DSI ORC absorbable haemostat is 100% regenerated and oxidized cellulose. It can be absorbed entirely in 7–14 days without altering the healing process. They have the ability to retain 35-40 times its own weight of blood and other bodily fluids. Moreover, according to their websites, spongostan absorbable haemostatic gelatin sponge, DSI ORC dental surgical sterile absorbable haemostatic sponge and I-sponge absorbable gelatin sponge also provide antibacterial protection that is efficient against a broad spectrum of gram-positive and gram-negative organisms, as well as antibiotic-resistant bacteria such as MRSA (Methicillin-resistant *Staphylococcus aureus*), VRE (Vancomycin resistant enterococci), and PSRP (Pneumococcal serine rich repeat protein).

#### 2.2.2 Nanoparticles used in sponges

In the pharmaceutical sector, nanoparticles (NPs) are widely used as a tool for research, diagnosis, and therapy. NPs are nanometric systems (liposomes, nanocapsules, dendrimers, inclusion complexes, micelles). Topical haemostatic medicines, such as thrombin and tranexamic acid, can promote and stabilise clot formation during bleeding. Inorganic minerals, such as kaolin, can also be applied to damaged blood vessels to stop the bleeding [131]. With the help of nanotechnology and polymer bioengineering, NPs-based synthetic sponges connect to activated platelets to enhance clotting in a safe and localised manner, leading them to aggregate more quickly to stop the bleeding. The following are some of the nanoparticles utilised in sponges to create antibacterial and wound-healing effects [132].

##### 2.2.2.1 Silver (Ag)Nanoparticles

**Fig. 8:**
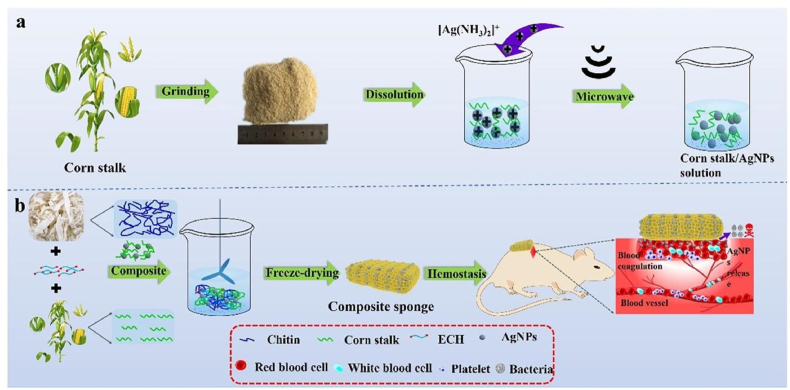
Chitin/corn stalk/Ag NPs composite sponge. **(a)** Depiction of a schematic for the preparation of Ag NPs. **(b)** Illustration of a schematic preparation of a chitin/corn stalk/Ag NPs composite sponge and the antibacterial haemostatic process [68]**.**

Silver nanoparticles, often known as AgNPs, are a great choice for improving the antibacterial characteristics of polymer-based sponges [133]. AgNPs can penetrate bacterial cell walls and exert antibiotic activity by releasing Ag^+^ [134]. AgNPs can thereby stop the development of biofilms, get rid of established biofilms, and counteract bacteria that is resistant to medication [135]. As a result of their application to highly specific surface areas, AgNPs can accomplish an effective sterilising effect at a lower concentration than other methods [136]. Sponges prepared with chitin (CH) and corn stalk (CS) were modified by adding silver nanoparticles (AgNPs) [68]. In an in-vivo rat liver injury scenario, the CH/CS and CH/CS/AgNPs sponges quickly absorbed the blood. The bleeding ceased in 31 and 30 s for CH/CS and CH/CS/AgNPs, respectively [68] as shown in Fig. 8, while the control group showed a haemostasis time of 120 s. CH/CS/AgNPs formed a strong physical barrier and provided pressure by rapidly absorbing plasma and expanding in volume throughout the haemostasis process. The physical barrier prevented the passage of a significant number of red blood cells and platelets, which resulted in the formation of an aggregation that was dense and robust. In addition, the wound that had been treated with the CH/CS/AgNPs sponges healed much quicker than the wounds that had been treated with the CH and CH/CS sponges [68].

In addition, the bactericidal activity of CS and CCS-AgNPs/CS sponges against *Escherichia coli* and *Staphylococcus aureus* was evaluated in a separate study by Huang et al., who developed a silver-loaded chitosan composite sponge with sustained silver release as a treatment that is effective for a prolonged amount of time against microbes. Compared to the CCS-AgNPs/CS sponge, which had a bacterial decrease of more than 98%, CS sponge exhibited a reduction of less than 17%. The excellent effectiveness against gram-positive and gram-negative *E. coli* was a direct result of the silver that was loaded into the sponges [137].

In conclusion, AgNPs do not provide haemostatic effect but have been employed in the sponges to offer antibacterial properties to the sponges. AgNPs are effective broad-spectrum antibacterial nanoparticles, hence promoting wound healing and decreasing the likelihood of infection.

##### 2.2.2.2 Silica (Si) and mesoporous Silica (MSNs) nanoparticles

**Fig. 9:**
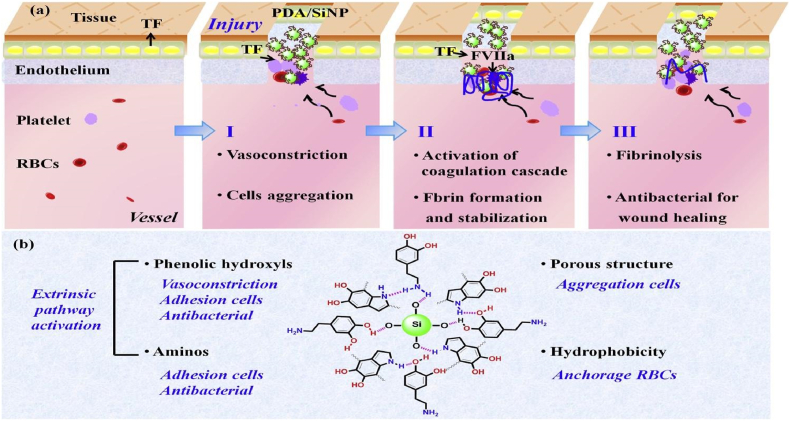
**Schematic diagram showing the haemostasis mechanism of PDA/SiNP. (a)** Formation of clots at the site of vessel injury after applying PDA/SiNP. **(b)** The potential haemostasis mechanisms of PDA/SiNP [139].

Numerous medical applications have made use of silica nanoparticles (SiNP) because of its large surface area and their ability to be tailored to a specific target. These includes drug delivery, molecular imprinting, and gene transfection. In order to stop bleeding, researchers here created a polydopamine-coated silica nanoparticle (PDA/SiNP) [138]. It has good degradability and antimicrobial activity as shown in Fig. 9. To induce a massive haemorrhage of vessels, femoral artery and vein injury was performed on SD rats. The recorded haemostatic time of the PSi4 group (PDA/SiNP with mass ratio of SiNP to oxidized DOPA at 5:1) was 104 ± 11 s, and the haemostasis time of the control group was 141 ± 9 s, which showed a better performance for the PSi4 group than the control group. Similarly, rat liver injury model was performed to investigate the haemostasis efficacy of PSi4. PSi4 had a rapid haemostasis time of 86 ± 3 s s, while the SiNP and blank groups took 155 ± 13 s and 137 ± 9 s, respectively. In a similar manner, PDA/SiNP applied to the wound site contracted blood vessels, directly lowering the volume of haemorrhage, and adhered to seal the wound [139].

Mesoporous Silica Nanoparticles (MSNs) are solid materials that are porous and have structures made of inorganic siloxane. They are becoming more popular and considered as a good choice for therapeutic applications. MSNs are also very effective at killing bacteria. They kill 99.80% of *Staphylococcus aureus* and 99.94% of *Escherichia coli* [140]. MSNs were used to prepare sponges with N-alkylated chitosan which showed better haemostatic efficiency [70]. According to the results of the platelet adhesion, APTT, TEG, and whole blood absorption tests, Glycerol-Modified N-alkylated Chitosan Sponges (MSN–GACS) has the potential to absorb a greater number of blood cells than the commercial product combat gauze. Haemostasis was accomplished in a rabbit femoral artery model in 69 ± 5.57 s for the MSN-GACS group and in 71 ± 5.57 s for the combat gauze (control group). Similarly, the MSN-GACS group achieved haemostasis in the rat liver injury model in 64.33 ± 3.06 s, which was much faster than the control group's time of 77.67 ± 4.73 s using the combat gauze. Results from H&E staining of liver tissues after MSN-GACS treatment showed that inflammation had been greatly decreased, and that the damage site and regenerated liver tissue were histologically indistinguishable from the original liver tissues. This demonstrated the favourable prognosis and low cytotoxicity of MSN-GACS. Because of their high porosity, their particular area of application, and the amount of silica they contain, these inorganic materials have a remarkable capacity to stop bleeding. MSNs could also help blood clotting to occur because they have a lot of silanol groups and negative net charges on their surfaces [141]. Thus, MSN-GACS demonstrated superior haemostatic effectiveness, better biocompatibility, and lower cardiovascular toxicity than combat gauze in the femoral artery model in rabbit and liver injury model in rat [70].

Generally, SiNP and MSN themselves do not have haemostatic effects. They have been incorporated into haemostatic sponges to enhance mechanical strength of the sponges and to provide antibacterial effect.

##### iii Zinc oxide (ZnO) nanoparticles

ZnO is an effective agent against a variety of diseases, including multi-drug-resistant organisms [142]. ZnO NPs are biocompatible and can be destroyed by the body over time, allowing an excess of zinc to be eliminated [143]. It is hypothesised that ZnO nanoparticles' generation of reactive oxygen species (ROS) contributes to the activation of pro-angiogenic proteins like vascular endothelial growth factor (VEGF) [144]. As a result of its antibacterial activity, high stability, photocatalytic activity and nontoxicity, ZnO nanoparticles were recently incorporated into a nanostructured antibacterial material composed of chitosan to create a topical haemostatic sponge [145,146]. Nanoparticle sponges made from chitosan, alginic acid and zinc oxide (CHI/AA/ZnO) were developed as a possible antibacterial biomaterial for bleeding control. Using a rabbit ear peripheral capillary haemorrhage model, the haemostatic effect was assessed, and the results indicated that the haemostatic time was shorter in the CHI/AA/ZnO group (93 ± 10.41 s) than in the CHI group (137 ± 24.75 s). *S. aureus* antibacterial activity was evaluated using disc diffusion and plate count procedures. The bacteriostatic impact of CHI/AA/ZnO samples was much higher than that of other samples. Thus, adding ZnO nanoparticles to hydrogel sponges can enhance tissue integration and wound healing [69].

Conclusions drawn from these studies indicates that ZnO NPs cannot develop the sponge on its own but when it is coupled with other composite materials to form a sponge, it can demonstrate wound healing properties.

## 3 Physical properties of sponges: their roles in haemostasis

Absorption of water, porosity, and mechanical qualities are essential characteristics of sponge materials, and these characteristics are strongly reliant on the underlying structure and morphology of sponges. According to Li et al., the porousness of a sponge is one of the most essential factors, since it may directly impact the amount of water it can absorb [147]. Sponge's highly porous nature makes it easier for them to absorb blood fluid, which in turn minimizes the amount of excess exudates [148]. Similarly, haemostatic chemicals and wound dressings rely on sponges' absorption properties. It relies greatly on the shape and structure of the sponges themselves [149]. Due to its relationship to absorption stability and ability to successfully bear the impact force of blood pressure, the mechanical characteristic of sponges is another crucial element in the development of haemostatic applications [45]. Among the haemostatic sponges mentioned in Table 2, some of the haemostatic sponges possess good mechanical strength and absorption capacity based on their parameters and in-vivo studies therefore indicating that they possess good haemostatic effects as well. Fan et al. claim that the addition of chitosan to cellulose sponges resulted in improved mechanical qualities, high water absorption capacity, and quick shape recovery [44]. Another study found that the inclusion of GO enhanced the mechanical and absorption capabilities of ACGS20, while AC has a low mechanical strength. A greater GO ratio in ACGS was associated with better blood coagulation [45].

Similar to this, Yuan et al. created an OBC/COL/CS composite sponge that demonstrated that the unmodified OBC had low mechanical strength, but that coupling CS into the OBC network increased the young's modulus and tensile strength, and that the strength even increased more after the addition of COL [55]. To treat noncompressible haemorrhages, Du et al. prepared MACS which exhibited good absorption and mechanical strength due to CS and the presence of hydrophobic alkyl chains [51]. According to Li et al., PVA/SA sponges demonstrated ultra-rapid water/blood absorption capacity compared to conventional sponges due to their high elasticity and microchannel structure but had lower mechanical strength due to their cylindrical structure. However, no noticeable damage was seen when applied at a pressure of 70%, demonstrating that these sponges could endure a high level of compression [66]. The SA/Gel sponge developed by Che et al. demonstrated a high rate of blood and water absorption as well as mechanical strength because the three-dimensional pore structure and connection between the pores in the haemostatic sponge were unaffected by the presence of polyelectrolyte multilayers [66]. KR-Sps sponges prepared by Yang et al. had good mechanical strength and absorption capacity due to the enhanced solid content and the ratio of SH and Nor [57].

Based on the physical qualities of the sponges described in the previous paragraphs, the enrichment of red blood cells and platelets generated a rapid and stable network structure, which operated as a physiological barrier for rapid haemostasis. As a result, it is essential to have a strong understanding of the micromorphology structure of the sponges. Haemostatic sponges with an interconnected and rich pore structure and high porosity can absorb more blood. In general, sponge pore diameter varied from 50 to 100 μm, and these pores facilitates the enrichment of both red blood cells and platelets [150,151]. The morphological structure of sponge is directly associated with the superior haemostatic efficacy and mechanical strength. The mechanical resilience of the sponges was measured using compression strain-stress curves. Sponge resilience can be evaluated using the elastic modulus. High values for tensile strain, tensile strength, and young's modulus shows sponge's ability to withstand mechanical stress, whereas low values suggest it may not withstand the stresses imposed by haemostatic applications [65,152,153].

## 4 Methodologies to evaluate haemostatic efficiency of sponges in-vitro and in-vivo

The two primary in-vitro tests used to evaluate the coagulation system nowadays are prothrombin time (PT) and activated partial thromboplastin time (APTT). Both tests are performed in vitro by combining coagulation factors and the time it takes for a clot to form. The coagulation cascade takes place on cell surfaces because coagulation is a spatially heterogeneous process without artificial mixing [154]. Several methods based on distinct functioning principles can be used to analyse the impact of sponges on the coagulation system and the haemostatic process. A combination of multiple methods is typically required to establish a conclusive result regarding each sponge type and their effects on haemostasis. Commonly employed methods are summarised below.

### 4.1 In-vitro methods

#### 4.1.1 Whole blood clotting test

In various clinical contexts and investigations, whole-blood coagulation assay is a technique used to monitor overall haemostatic status. These global haemostasis tests can be valuable, especially when a quick turnaround is required. They can be useful in determining if a patient is bleeding due to coagulopathy or an anatomic reason [155]. After mixing blood with sodium citrate to prevent clotting, sponge samples were weighted and incubated with the blood at 37 °C. As a blank control, blood was utilised without any samples. CaCl_2_ was then added to initiate coagulation, test tubes were examined at regular intervals and the clotting time was noted [42,46]; X [47].

#### 4.1.2 APTT (activated partial thromboplastin time) and PT (prothrombin time) test

In the clinical setting, blood clotting tests are evaluated using the activated partial thromboplastin time (APTT) and the partial thromboplastin time (PT). The partial thromboplastin time (PTT) is used to test the intrinsic system (factors VIII, IX, XI, and XII) and the common pathways (factors V and X, prothrombin, and fibrinogen). In order to initiate clotting via the intrinsic pathway, a phospholipid platelet substitute is administered to the patient's blood. The addition of kaolin or contact activator transforms the test into an “activated PTT” (APTT) [156]. With the help of an activator (such as silica or ellagic acid), APTT can reduce the reference range to 30–40 s by speeding up the clotting process [157]; X [47]. The APTT test reveals how long it takes for blood to clot when body tissue or blood vessel walls have been damaged. Blood proteins known as “clotting factors” normally assemble in a specific order when a blood vessel is ruptured to produce blood clots that immediately stop bleeding. The APTT is a more sensitive version of the PTT, and it is used to track how a patient responds to heparin therapy [158].

#### 4.1.3 Platelet adhesion/activation

Platelet adhesion, activation, and aggregation are all main events in the haemostasis and thrombosis [159]. Centrifuging whole blood (WB) produced platelet-rich plasma (PRP) (1200 rpm, 10 min). A lactate dehydrogenase (LDH) kit was utilised for the quantitative investigation of platelet adhesion. Basically, using an LDH kit, the LDH activity of platelets was measured. Using a standard curve for OD at 440 nm, the number of adherent platelets was calculated. Each sample was submerged in 2 mL of platelet-rich plasma (PRP) for predefined periods (i.e., 15, 30, 60, 120, 180 and 300 s) and incubated for 30 min at 37 °C. Following three PBS rinses to remove non-adherent platelets, the samples were treated for an hour at 37 °C with PBS containing 1% polyethene glycol phenyl octyl ether to dissolve the platelets. Therefore, based on the standard curve, the absorbance of the supernatant was utilised to determine the number of platelets [160].

Erythrocyte aggregation is critical in the pathophysiology of blood circulation, and it occurs by electrostatic force and macromolecule bridging. Rouleaux are created as a result, which later transforms into spherical objects of a consistent size [161]. To understand the sample's effect on erythrocyte adherence, the morphological alterations of erythrocytes were observed after adhesion. The coagulated blood clots were taken out, cleaned with PBS to get rid of non-adherent erythrocytes, and preserved with glutaraldehyde. Using ethanol, the fixed blood clots were dehydrated, and the samples were freeze-dried after tertiary butanol was sprayed. Subsequently, SEM was used to examine erythrocyte adherence to the samples (X [47].

#### 4.1.4 Flow cytometry analysis (FACS)

In order to investigate the function of sponges in platelet aggregation, platelets from different species (rat, mouse, rabbit) were analysed using flow cytometry to determine which ones reacted with Alexa 488-fibrinogen. The samples were treated with Platelet-Rich Plasma (PRP), incubated, and finally rinsed with phosphate-buffered saline (PBS). Next, the samples were discarded. The leftover liquid was centrifuged to remove the supernatant. PBS and *anti*-P selectin antibodies labelled with APC were added and incubated. Then platelets were resuspended with PBS. Flow cytometry was finally used to measure CD62p levels. The same method was utilised to evaluate the control group (no sample) [45].

#### 4.1.5 Thromboelastography (TEG) analysis

TEG was utilised to compare the clotting activities of materials in vitro [162,163]. Blood clot strength was evaluated over time during the clotting process. Dried samples (sponges) were pulverised finely before being transferred to a centrifuge tube. Sodium citrate anticoagulated whole blood was often made by mixing blood extracted from the animals' hearts (rabbits, mice, and rats) with an anticoagulant containing sodium citrate. 1 mL of anticoagulated whole blood was added to each sample. After mixing, it was transferred to the test cups, followed by the CaCl_2_ solution for testing [164]; X [47].

### 4.2 In-vivo methods

#### 4.2.1 Haemostatic evaluation in-vivo

In-vivo haemostatic validation of sponges was performed using animal models such as the tail transect model (X. [43,64,72], femoral artery and vein incision model [59,164], liver trauma model [42,64] and pig model of traumatic coagulopathic bleeding [48] The description of these animal models is elaborated below.

Tail amputation models in mice and rats have been widely employed. Initially, 10% chloral hydrate/sodium pentobarbital (0.3–0.5%) was used to induce anaesthesia in this model. Afterwards, 2–5 cm was effectively cut off the end of their tails in rats whereas in mice model the tail was cut off from the middle part. Haemostatic sponges were placed on the wound, and blood loss and the time it took for the wound to clot were recorded (X [43,64,72].

A similar femoral artery and vein incision procedure was carried out on rats to evaluate the level of haemostatic efficiency provided by various sponges. In this approach, the initial step was administering 10% chloral hydrate to rats via the intramuscular route. Then, the leg hair of the rats was shaved, and the thigh muscle of the rats was opened. A sponge was then applied to the bleeding area, and the amount of time it took for the wound to stop bleeding as well as the amount of blood loss was recorded [59,64]. Femoral injury model was also created in. After anaesthesia, a longitudinal incision approximately 5 cm in length was produced. After the femoral artery and vein were made accessible, they were both dissected free from their adjacent tissue. Following the application of a light with constant pressure, haemostatic was placed on the wound. The haemostatic time and blood loss were recorded [59,164].

In rat liver injury models, rats were first put under anaesthesia and then had their chests opened so that a cut measuring one and a half cm could be made in the right lobe of the liver. Immediately following the dissection of the liver, sponges were placed on the injury, and the haemostatic time and blood loss was noted. A procedure quite similar to the one described above was also carried out on rabbit. In this model a scrape on the anterior lobe of the liver that measured 0.5 cm in length, 0.5 cm in depth, and 0.2 cm in width was performed. After the bleeding started, a haemostatic sponge was applied to the wound to stop the bleeding and both haemostatic time and total volume of blood loss were reported [42,64].

Traumatic liver haemorrhage model was created in haemodilutional coagulopathic pigs. In this model, warm normal saline solution was given at 4 ml/kg per hour by peripheral catheterization into the ear vein. The right femoral artery was catheterized for mean arterial pressure (MAP) monitoring, blood sampling, and blood extraction. To limit fluid compensation, a laparotomy was performed to expose the liver, followed by a splenectomy. By exchanging 40% of the anticipated total blood volume with a cold normal saline solution (4 °C) (3 times the extracted blood and spleen), the animals were hemodiluted. An acrylic plate was used to support a liver lobe thicker than 3 cm, and a plastic bag was placed underneath to catch any blood that could spill out. Hepatic damage was generated by the fall of a stainless-steel ball (512 g, d = 5 cm) through a transparent acrylic tube from a height of 50 cm. Haemostatic time was recorded after the bleeding site was covered with a sponge [48]*.*

## 5 Discussion and summary

The demands on the performance of haemostatic sponges are becoming more stringent as medical services continue to evolve. The ability to produce innovative haemostatic sponges that are rapid, efficient, safe, and ready to use is critical. For the purposes of this review, we have looked at composite materials (natural polymers and synthetic polymers) used to construct sponges, the haemostasis mechanism, and methods for determining the haemostasis efficacy of sponges in both in-vitro and in-vivo. Several in-vivo models, such as tail amputation model, liver trauma model, and femoral artery model, are utilised to examine the haemostatic efficacy of sponge in rats, mice, rabbits, and pigs. Haemostatic sponges made from various composite materials have a high-water absorption capacity, porosity, and high mechanical resistance. The developed sponges also have good flexibility and good adhesion to wounds, thus being employed to treat non-compressible haemorrhages. According to the physical parameters covered in this review, high porosity and water absorption capacity are correlated with rapid blood absorption, blood cell entrapment, and subsequent clot formation at the bleeding site. Similarly, the mechanical qualities of a sponge are directly connected to its capacity for absorption, because sponges with good mechanical properties can efficiently resist the impact of blood pressure. The key characteristic of an extraordinary haemostatic substance is the capacity to reduce bleeding time effectively, as demonstrated in this review by the fact that different sponges have various bleeding times. These sponges have different properties, such as their unique blood clotting times and impacts on the healing process. Natural polymers like chitosan, cellulose, gelatin, silk fibroin, graphene oxide, alginate, and hyaluronic acid have been widely used as a substrate for haemostatic sponges due to their advantageous qualities. These substances also contained active medicinal components of various pharmacological properties.

As a result of non-toxicity and biocompatibility, chitosan sponges have been extensively used as a haemostatic sponge material. Examples of chitosan's haemostatic properties include the accumulation of red blood cells (RBCs), platelet activation (PLT), and the contact activation pathway. Chitosan is an appealing biomaterial for wound care due to its biocompatibility and inherent haemostatic and antibacterial capabilities. Researchers have also observed that chitosan sponges can induce spontaneous agglutination of blood, thrombin production, and the formation of new tissue for healing. Likewise, other cellulose-based sponge derivatives have been developed, each with their own mechanisms and limits. The cellulose composite sponge absorbs water at a rapid rate, allowing it to swiftly absorb and concentrate blood, increasing blood cell adhesion and aggregation, and meeting blood coagulation goals. Cellulose-based sponges increase platelet aggregation and blood coagulation simply by contact activation. Similarly, studies on gelatin sponges revealed that they exhibit weak mechanical strength but can be improved by crosslinker. They have no inherent haemostatic effect but generate haemostasis due to their porous shape and that when the sponges fill with blood, platelets come into close contact and begin to agglomerate, triggering a clotting cascade. As a result, the haemostatic action of the gelatin sponge is not optimal for people with impaired coagulation processes since they rely on the participation of body coagulation factors to stop bleeding. Similarly, gelatin's most significant drawback is that it has a low mechanical strength and a low resistance to hydrolysis. However, these properties can be enhanced with the application of crosslinking agents. Other composite sponge materials with comparable haemostatic and antibacterial properties can also be used as haemostatic materials.

Graphene oxide has a strong affinity for water due to its oxygen functional groups, which enables it to absorb plasma fast, concentrate blood cells, platelets, and coagulation factors, and speed up the coagulation. Similarly, alginate can absorb a lot of wound exudates and create a physiologically moist environment that aids in wound healing. Hyaluronic acid has high water affinity due to its oxygen functional groups, which can quickly absorb plasma, enrich blood cells, platelets, and coagulation factors, and speed up coagulation. Furthermore, starch-based sponges induce thrombosis and reduce coagulation time, while silk protein sponges act as carriers for fibrinogen and/or thrombin maintaining their haemostatic properties. In addition, due to their capacity to counteract the drawbacks of natural polymers, synthetic polymers are gaining popularity for use in haemostasis. Materials made from synthetic polymers (i.e., polythene glycol, poly vinyl alcohol) are commonly used as physical barriers because of their high adherence to tissue and histocompatibility. This is due to the fact that these substances have low toxicity, excellent tissue adherence and can serve as carriers for therapeutic drugs. On the other hand, sponges made of nanoparticles are largely bacteriostatic and can promote wound healing. Among all the nanoparticles, MSNs possess outstanding haemostatic efficiency due to their high porosity, specific area of application, and silica composition. There is a plethora of commercial sponges available in the market that can soak up bodily fluids. These sponges include porous networks of varying diameters. These sponges are used to stop bleeding from the body's capillaries, veins, and smaller arteries during a wide range of surgical procedures.

Despite significant development in the field, additional research is needed as the underlying mechanisms of all haemostatic sponges have not been adequately investigated and comprehended. Testing methods and animal models utilised in the studies reviewed here vary significantly, thus comparison of the haemostasis effectiveness of the developed sponges is not easy. Methods could be standardised to facilitate comparison and promote proper development in the field. In some conditions, the removal of non-absorbable haemostatic materials may result in re-bleeding in some patients. To avoid this problem in the future, degradable and/or absorbable sponges should be developed. Also, haemostatic sponges may be constructed to target tissue wounds with varying forms and depths. Developing effective haemostatic devices, designing, and improving animal models of trauma, and conducting clinical trials in multidisciplinary settings are all necessary for conducting robust studies to produce optimal haemostatic sponges.

## Ethics approval

This is a review paper so there is no ethics approval.

## Declaration of competing interest

The authors declare that they have no known competing financial interests or personal relationships that could have appeared to influence the work reported in this paper.

## Abbreviation list

AA: Alginic Acid
AC: Alkylate Chitosan
Ag: Silver
ALG: Alginate
APTT: Activated Partial Thromboplastin Time
BpB: Blood Protein Barrier
BSP: Bletilla Striata Polysaccharide
BGCS: Bletilla Striata Polysaccharide/Graphene Oxide Composite Sponge
BNC: Bacterial Nanocellulose
CS: Chitosan
CS/CHT/CHI: Chitosan
Cel: Cellulose
CHI–C: Catechol Conjugated Chitosan
COL: Collagen
CNF: Cellulose Nanofibers
CNC: Cellulose nanocrystals
CMC: Carboxy Methyl Cellulose
CGS: Cross linked graphene oxide
CSCA: Capric Acid Modified Chitosan
CF: Collagen Fiber
CSP: Corn Stalk Pith
CT: Chitin
Ce: Cerium
CS: Corn Stalk
CMC/SA: Carboxymethyl Chitosan and Sodium Alginate
CG/OF: Oxidized Cellulose Fibers
DB: Diatom Bio silica
Dex-PDM: Cationic dextran-d poly (2-dimethyl amino)-ethyl methacrylate)-grafted dextran
DFO: Desferioxamine
EDC: 1-ethyl-3-(3-dimethylaminopropyl)
EDC: 1-ethyl-3-(3-dimethyl aminopropyl) carbodiimide
eMPs: Electro spraying Microspheres
EW: Egg White Protein
FACS: Flow Cytometry Analysis
Gel/G/Gp: Gelatin
GTA: Glutaraldehyde
GP: Genipin
GDL: d-glucono-d- lactone
GO: Graphene Oxide
HA: Hyaluronic Acid
KR-Sp: KR12 Immobilized Starch Based Sponge
MACS: Micro Channelled Alkylated Chitosan Sponge
MAP: Mean Arterial Pressure
mTG: microbial transglutaminase
MMT: Montmorillonite
MBG: Mesoporous Bioactive Glass
MS: Mesoporous Silica
MN-GACS: Glycerol Modified N-Alkylated Chitosan Sponge
NHS: N-hydroxy succinimide
NPs: Nanoparticles
OBC: Oxidized Bacterial Cellulose
OS: Oxidized starch
ODs: Oxidized Dextrans
OBNC: Oxidized Bacterial Nanocellulose
PEG: Polyethylene Glycol
PVP: Polyvinylpyrrolidone
PVA-CS: Poly-vinyl alcohol-chitosan sponge
PAA: Poly (acrylic acid)
PVA: Polyvinyl Alcohol
PDA: Polydopamine
PT: Prothrombin Time
PBS: Phosphate Buffered Saline
PRP: Platelet Rich Plasma
PLT: Platelet Activation
QCs: Quaternized Chitin Derivatives
RBC: Red Blood Cell
rhcolIII: Humanized type III collagen recombinant protein
RES: Resveratrol
SSAD-CS: Skin Secretion of Andrias Davidians Cellulose Sponge
S-CS/TPS: Chitosan-Tilapia Peptide Microsphere Sponges
St: Starch
SF: Silk Fibroin
SA: Sodium Alginate
STMP: Sodium Trimetaphosphate
Si: Silica
SEM: Scanning Electron Microscopy
TA: Tannic Acid
TOCN: TEMPO Oxidized Nanocellulose
TEMPO: (2,2,6,6-Tetramethylpiperidin-1-yl) oxyl
Th: Thrombin
TA: Tranexamic Acid
TEG: Thromboelastography
ZnO: Zinc Oxid
